# Targeting Macrophage Polarization for Reinstating Homeostasis following Tissue Damage

**DOI:** 10.3390/ijms25137278

**Published:** 2024-07-02

**Authors:** Qiran Du, Anna Dickinson, Pruthvi Nakuleswaran, Susan Maghami, Savindu Alagoda, Andrew L. Hook, Amir M. Ghaemmaghami

**Affiliations:** 1 Immuno-Bioengineering Group, School of Life Sciences, University of Nottingham, Nottingham NG7 2RD, UK; qiran.du@nottingham.ac.uk; 2 Medical School, Faculty of Medicine and Health Sciences, University of Nottingham, Nottingham NG7 2RD, UK; mzyad17@nottingham.ac.uk (A.D.); mzypn3@nottingham.ac.uk (P.N.); mzysa40@exmail.nottingham.ac.uk (S.A.); 3 Hull York Medical School, University of York, York YO10 5DD, UK; hysm47@hyms.ac.uk; 4 School of Pharmacy, University of Nottingham, Nottingham NG7 2RD, UK; andrew.hook@nottingham.ac.uk

**Keywords:** macrophage polarization, immune modulation, biomaterials, tissue repair, inflammation, medical devices, immune-instructive materials

## Abstract

Tissue regeneration and remodeling involve many complex stages. Macrophages are critical in maintaining micro-environmental homeostasis by regulating inflammation and orchestrating wound healing. They display high plasticity in response to various stimuli, showing a spectrum of functional phenotypes that vary from M1 (pro-inflammatory) to M2 (anti-inflammatory) macrophages. While transient inflammation is an essential trigger for tissue healing following an injury, sustained inflammation (e.g., in foreign body response to implants, diabetes or inflammatory diseases) can hinder tissue healing and cause tissue damage. Modulating macrophage polarization has emerged as an effective strategy for enhancing immune-mediated tissue regeneration and promoting better integration of implantable materials in the host. This article provides an overview of macrophages’ functional properties followed by discussing different strategies for modulating macrophage polarization. Advances in the use of synthetic and natural biomaterials to fabricate immune-modulatory materials are highlighted. This reveals that the development and clinical application of more effective immunomodulatory systems targeting macrophage polarization under pathological conditions will be driven by a detailed understanding of the factors that regulate macrophage polarization and biological function in order to optimize existing methods and generate novel strategies to control cell phenotype.

## 1. Introduction

Tissue damages following accidental (e.g., cuts and burns) or therapeutic incidents (e.g., surgical implantation of medical devices) trigger tissue repair mechanisms that are broadly similar and consist of several phases, including coagulation, inflammation, immune cell infiltration, and proliferation, inflammation resolution, tissue angiogenesis and remodeling, as well as scar formation, which requires an orchestration among different cell types [1,2,3]. In the first few hours of tissue injury, guided by chemokines, neutrophils rapidly migrate to these sites, engulf the invading pathogens, and release various immune mediators, such as cytokines, chemokines, growth factors, and enzymes. Infiltrating monocytes are activated and differentiated into macrophages which are essential in maintaining tissue homeostasis by regulating the initiation and resolution of inflammation and tissue repair [3,4,5]. In this process, macrophages have three main functions, including phagocytosis, antigen presentation, and secretion of immune modulators [6,7,8]. As recent studies have shown, macrophages can mobilize fibroblasts, mesenchymal stem cells, epithelial cells, and endothelial cells by producing these immune modulators to promote tissue regeneration and wound healing [3,9].

Macrophages are strategically located in various tissues and exhibit significant plasticity in response to different microenvironmental changes [10]. After being activated by cytokines or other danger signals, macrophages can acquire a spectrum of functional phenotypes that are represented by pro-inflammatory M1 (classically activated) and anti-inflammatory M2 (alternatively activated) macrophages at either end of this spectrum. M1 macrophages mainly secrete pro-inflammatory molecules to eliminate invading pathogens and boost inflammation, while M2 macrophages express various anti-inflammatory molecules that support tissue regeneration and remodeling [7,11,12]. It is worth noting that continuous activation of M2-like macrophages could lead to fibrosis which is the outcome of dysregulated M2 activation [13,14]. However, in damaged tissue, macrophage phenotypes are not a single homogenous population, with cells exhibiting both M1 and M2-like characteristics being present [7,15,16]. The emergence of this heterogeneous cell population can be affected by different stimuli, and there remains a significant number of unidentified factors that contribute to a specific spectrum of macrophage-activated states [5]. However, it is crucial for the macrophage population to be correctly regulated as the outcomes can vary from tissue regeneration to chronic inflammation and ultimately tissue damage [3,7,17,18]. Interestingly, this endogenously regenerating capability diminishes with age and a range of pathologies (e.g., foreign body response, non-healing wounds) which may develop into chronic immune responses and fibrosis or even cancer [15,19]. For example, in diabetes, dysfunctional macrophages disrupt the natural progression from the inflammatory to the repair and remodeling stage in wounds. This disturbance results in the maintenance of an inflammatory environment, hindering the infiltration and operation of pro-healing immune cells [20]. The recognition of macrophages in regulating these processes has underpinned the emergence of many approaches focused on rebalancing immune homeostasis to support tissue repair by targeting macrophage polarization to enhance immune-mediated tissue regeneration [17,21,22,23,24,25,26,27,28,29,30].

Therefore, it is necessary to understand the factors affecting macrophage polarization and biological functions and review the applications targeting macrophage polarization in the management and treatment of various stages of tissue damage and wound healing to provide opportunities for the rationale design of better therapies. This review will address the functions of macrophages and modulatory strategies in regulating macrophage phenotypes in response to tissue damage with a focus on using biomaterial-based strategies. Initially, an overview of the biological role of macrophages, including macrophage origins, activation, phagocytosis ability, antigen presentation activity, and immune modulator production involved in macrophage polarization was provided [6,7,8,10,11,31,32,33,34,35,36,37,38,39,40,41]. Then we discussed different strategies, including drug delivery and biomaterial-based approaches to target macrophage phenotypes therapeutically for the treatment of different inflammatory diseases and cancer therapy [17,20,21,22,23,24,25,26,27,28,29,30,42,43,44,45].

## 2. Macrophage Biological Roles

### 2.1. Macrophage Origins

**Figure 1:**
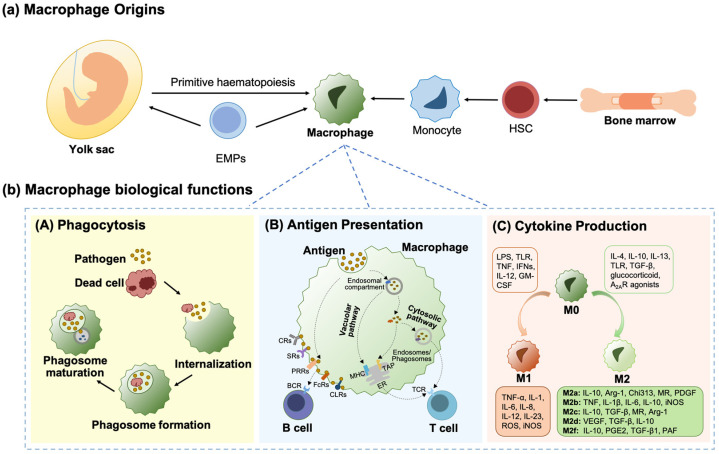
Schematic diagram of macrophage origins and biological functions. (**a**) Macrophage origins: macrophages derived from the bone marrow and yolk sac [36]; (**b**) Macrophage biological functions. (**A**) Phagocytosis: this function is to recognize and degrade pathogens, dead cells, and cell debris under the mediation of complex receptors and the activation of the actin-dependent mechanism, including four phases: (1) targeting particles detection; (2) internalization process activation; (3) phagosome formation; (4) phagosome maturation [53,54]; (**B**) Antigen presentation: B cells can recognize antigens through the variable domain of BCR, while T cells exclusively detect a wide range of antigens presented by MHC molecules macrophages through TCRs via cross-presentation, including the cytosolic pathway and vacuolar pathway [8,55,56]; (**C**) Cytokine production: M1-like macrophages release pro-inflammatory cytokines, such as IL-1 and TNF-α, whereas M2-like macrophages mainly produce anti-inflammatory cytokines, such as IL-10 and TGF-β [40,57,58]. Abbreviations: A2AR, adenosine A2A receptors; Arg-1, arginase-1; BCR, B cell receptor; Chi3l3, chitinase3-like protein 3; CLRs, C-type lectin receptors; CRs, complement receptors; EMPs, erythro-myeloid progenitors; ER, endoplasmic reticulum; FcRs, Fc receptors; GM-CSF, granulocyte macrophage-colony stimulating factor; HSC, hematopoietic stem cells; IFNs, interferons; IL, interleukin; iNOS, inducible nitric oxide synthase; LPS, lipopolysaccharides; MHC, major histocompatibility complex; MR, mannose receptor; PAF, platelet-activating factor; PDGF, platelet-derived growth factor; PGE2, prostaglandin E2; PRRs, pattern recognition receptors; ROS, reactive oxygen species; SRs, scavenger receptors; TAP, transporter associated with antigen processing; TCR, T cell receptor; TGF-β, transforming growth factor-β; TLR, Toll-like receptor; TNF, tumor necrosis factor; VEGF, vascular endothelial growth factor.

Russian naturalist Ilya (Elie) Metchnikoff first identified macrophages in starfish larvae in 1882. By combining the evolutionary and ontological views Metchnikoff developed a novel understanding of cell-mediated immunity and made the initial discovery of the macrophage phagocytosis mechanism [33,36,46]. Macrophages are the crucial defenders against invasive pathogens because of their phagocytosis ability which originated in conserved phagocytes over 500 million years ago [21,36,37]. Van Furth and Cohn proposed in 1968 that circulating blood monocytes are the source of all tissue macrophages [35], but later on, fetal embryonic yolk sack was proposed as the precursor of tissue-resident macrophages [47,48]. It is now well-established that the origin of monocyte-derived macrophages in different organs (such as the liver, heart, and dermis) is hematopoietic stem cells (HSC) in the bone marrow during the neonatal period [49,50], whereas, macrophages derived from yolk sac have potentially three sources: (1) primitive hematopoiesis producing the so-called primitive macrophages; (2) ‘early’ erythro-myeloid progenitors (EMPs) giving rise to pre-macrophages that develop into macrophages in different embryonic tissues; (3) ‘late’ EMPs migrating to fetal liver and subsequently differentiating into fetal liver monocyte and then resident macrophages in all developing organs (except the brain), (Figure 1) [31,34,47,50,51]. Interestingly, resident macrophages derived from monocytes are terminally differentiated and have no self-renewal ability, whereas embryo-derived cells retain self-renewal potential [52].

When Metchnikoff first defined macrophages in the starfish, because starfish have no formal vascular system [59], he argued that the inflammation was independent of vessels, which countered the German pathologist Julius Friedrich Cohnheim’s claims: there is no inflammation without blood vessels [60]. However, it is now clear that macrophages are involved in inflammation and tissue repair through mechanisms that could be dependent or independent of monocyte recruitment from the vasculature [61,62].

Macrophage functions can be mainly divided into three parts: (1) phagocytosis of invading pathogens, microorganisms, apoptotic cells, and cell debris [53]; (2) antigen presentation to present processed antigens to B cells and T cells [56]; (3) secretion of immune mediators, including chemokines, cytokines, growth factors, antibodies, enzymes, as well as inducible nitric oxide synthase (iNOS) and reactive oxygen species (ROS), (Figure 1) [5,40]. Moreover, macrophages participate in tissue remodeling and their dysregulation plays an important role in the progression of various inflammatory diseases and cancer [5].

### 2.2. Macrophage Phagocytic Ability

Phagocytosis is a primary function of macrophages and has evolved to eliminate infectious agents and cell debris as well as prevent the release of potentially immunogenic and toxic contents from pathogens and apoptotic cells into surrounding tissue during the resolution of inflammation and tissue remodeling [5,63]. Phagocytosis mostly involves the recognition and degradation of particles larger than 0.5 µm and can be divided into four phases: (1) detection of targets (2) internalization process (3) phagosome formation (4) phagosome maturation, (Figure 1) [64,65]. Tissue-resident macrophages can detect microenvironmental changes (e.g., hypoxia), recognize pathogen-associated molecular patterns (PAMPs), such as lipoteichoic acids and formylated peptides in bacteria and mannans in the yeast, and damage-associate molecular patterns (DAMPs) produced by damaged host cells through antigen-specific cellular receptors (including pattern recognition receptors (PRRs)) [66]. Meanwhile, in the early stage of apoptosis, damaged cells emit “find me” signals, including lysophosphatidylcholine (LPC) [67] or phosphatidylserine (PtdSer) [68], chemokine C-X3-C motif ligand 1 (CX3CL1) [69,70], sphingosine-1-phosphate (S1P) [71], and nucleotides [72], such as ATP and UTP [73], to attract macrophages [74].

**Figure 2:**
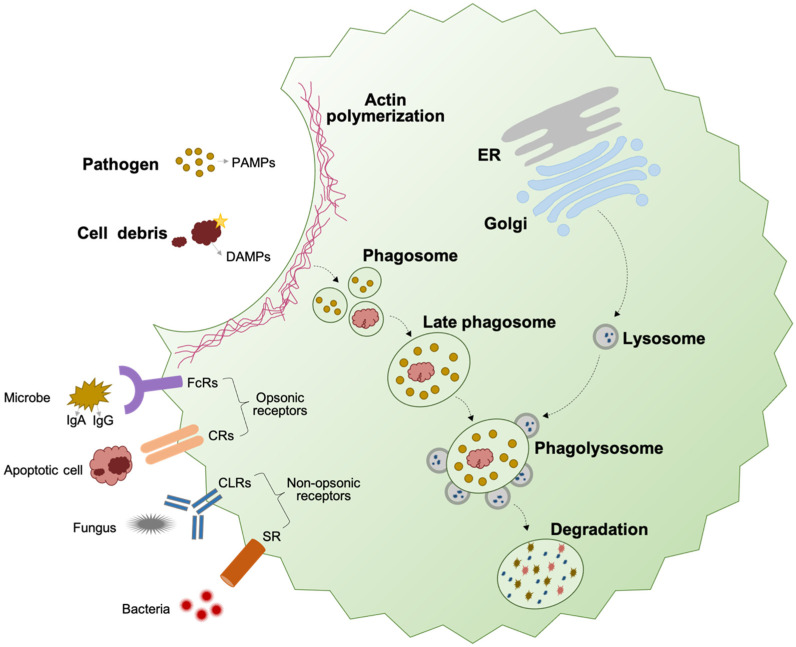
Phagocytic mechanisms of macrophages. Several processes are involved in macrophage phagocytosis, such as phagocytosis receptor-ligand binding, actin polymerization, internalization of particles, as well as phagosome formation and degradation [53,54,63]. Abbreviations: CLRs, C-type lectin receptors; CRs, complement receptors; DAMP, damage-associate molecular patterns; ER, endoplasmic reticulum; FcRs, Fc receptors; IgA, immunoglobulin A; IgG, Immunoglobulin G; PAMP, pathogen-associated molecular patterns; SRs, scavenger receptors.

The particle Internalization takes place through specific surface plasma-membrane receptors, which can be divided into two types, including opsonic receptors and non-opsonic receptors, (Figure 2). Opsonic receptors, such as Fc receptors (FcRs) and the complement receptors (CRs) [75], can recognize host-derived proteins called opsonins (e.g., mannose-binding lectin, fibronectin, and milk fat globulin (MFG-E8)) bound to particles to be ingested [76,77]) [64]. However, non-opsonic receptors, including scavenger receptors (SRs) (e.g., SR-A, CD36, as well as stabilin 1 and 2) [78,79,80,81], C-type lectin receptors (CLRs) (e.g., mannose receptor (MR, CD206), Mincle, Dectin-1 and 2, and MCL) [82,83], and lectin-like recognition molecules (e.g., CD33 and CD169) [83], are able to directly identify molecular patterns on target particles [64]. Other receptors, such as T cell immunoglobulin and mucin domain-containing molecule 4 (TIM-4) [84], brain angiogenesis inhibitor 1 (BAI1) [85], tyrosine-kinase-activated receptors (e.g., tyrosine-protein kinase receptor (Tyro3), AXL receptor tyrosine kinase (Axl), and myeloid-epithelial-reproductive tyrosine kinase (MERTK)) [86], and their ligands, such as growth-arrest-specific-6 (Gas 6) and Protein S (PROS1) are also involved in phagocytosis process to detect apoptotic cells [87].

Upon covering the particle, the membrane of macrophages closes at the distal end and creates the phagosomes through F-actin depolymerization to form the early phagosome with particle internalization, (Figure 2) [88]. Subsequently, this early phagosome and its contents mature to become a late phagosome through sequential fusion and fission events, and ultimately it fuses with lysosomes to form a phagolysosome under the regulation of annexins, as well as proteins associated with the soluble N-ethylmaleimide-sensitive factor attachment protein receptors (SNAREs) mechanism of membrane fusion, such as synaptobrevin and syntaxin [89,90,91]. This process is known as the phagosome maturation [92,93]. The phagocytosis in macrophages is a complex process, involving the activation of various receptors and downstream signaling pathways to promote actin polymerization and particle internalization [54,63,64,94]. Some complex particles, such as bacteria and invasive fungi, have the capacity to simultaneously interact with multiple receptors in intricate and unpredictable ways. To avoid being recognized, some bacteria change their surface antigenic properties to non- or poorly recognizable patterns, such as a modified version of Lipid A [30,38,39,40]. If still recognized, they can try to avoid phagocytosis by increasing their size, as macrophages prefer to engulf smaller targets. For example, various species of Mycobacteria and the yeast C. neoformans have been demonstrated to increase their size in order to evade phagocytosis [41]. If the bacteria are unable to escape phagocytosis, they may still attempt to survive by becoming tolerant to acidity inside the phagosome [42]. Once these microorganisms have successfully evaded phagocytosis or destruction, they can exploit macrophages as a protective niche against other immune cells that exhibit higher levels of microbicidal activity, or as a temporary living place where they can proliferate, grow, or germinate prior to the non-lytic exit from the host cells and dissemination to other cells and tissues [95]. During this process, living bacteria can also modify the signaling pathways in macrophages to prevent the infected cells from undergoing apoptosis and further allow their maximal multiplication. For instance, *Legionella pneumophila* can produce the type IV effector protein SidF into the macrophage cytosol, targeting the proapoptotic factor BNIP3, thereby inhibiting mitochondria-mediated apoptotic signaling [96].

During phagocytosis, macrophages can remarkably increase the area of their membrane by up to around 300% [85,86]. There are two different sources for providing membranes to envelop the target particles: intracellular vesicles and granules and folds in the plasma membrane [87]. The mobilization of these membrane reservoirs during the phagocytosis of large particles may need a trigger, and the plasma membrane itself may act as a mechano-chemical tuning mechanism by creating membrane tension during the uptake [88]. The total amount of extra membrane that can be mobilized for phagocytosis may be the major physical constraint limiting the maximum phagocytic capacity of macrophages [89]. This is supported by the study using a multiparametric and high-throughput assay, which examined the impact of target size (liquid lipid droplets and solid polystyrene beads) on the phagocytic uptake of RAW 264.7 murine macrophages. The findings indicated that the primary factor limiting particle uptake, irrespective of size or nature, is the total surface area engulfed by the cell [90].

Therefore, particle sizes that cells can ingest have an upper limit [91,92]. When the particles exceed this limit to be too large to be encompassed by macrophages or other immune phagocytes, it causes stalling of the phagocytic process, which is called frustrated phagocytosis [91,92]. This limit depends on the particle materials and geometry, as well as the cell type [91]. For spherical particles, an in vitro study comparing the effects of the size and surface properties of the polystyrene microspheres for phagocytic uptake by alveolar macrophages indicated that polystyrene microspheres with diameters more than 10 μm were not engulfed by alveolar macrophages [93]. Whereas, in the other study optimizing conditions for efficient phagocytosis of rifampicin-loaded poly(lactic-co-glycolic acid) (PLGA) microspheres by alveolar macrophages, results showed that macrophages could take up 10 μm particles, although compared with the large particles, particles with the size between 1 μm and 6 μm were engulfed by more macrophages [94]. For needle-shaped particles, this limit is typically around 15 µm [91,95,96], but there are also other studies showing that particles longer than 15 µm can also be internalized. For instance, THP-1 macrophages and primary murine peritoneal macrophages could internalize 20 µm long calcium carbonate (CaCO_3_) needles (aspect ratio > 20) in the study of investigating the impact of CaCO_3_ particle shape on phagocytosis and pro-inflammatory response in differentiated THP-1 macrophages [97]. For fibers, this limit is around 20 µm. Long fibers, such as asbestos and carbon nanotubes cannot be engulfed by macrophages, hence resulting in frustrated phagocytosis [95]. For example, exposure to asbestos during mining and in industry led to a global pandemic of lung diseases [96]. Toxicologists have developed a paradigm in which a hazardous fiber is longer than around 20 µm, thinner than 3 µm, and bio-persistent in the lungs, in other words not dissolving or breaking into shorter fibers [98]. Apart from asbestos, other crystals or crystalline particles, such as uric acid crystals, alum, and silica, can cause frustrated crystal phagocytosis at the cell surface by rupturing and damaging the lysosome and releasing some of their contents, including cathepsin B, into the cytoplasm, which promotes the production of ROS and further triggers inflammation, fibrosis, and oncogenesis [95,99,100,101].

### 2.3. Macrophages as Antigen-Presenting Cells

Antigens engulfed by macrophages are typically degraded in lysosomes after phagosome maturation allowing altered antigens to be presented to B cells and T cells at the cell surface [97]. While B cells are able to recognize membrane bound (e.g., provided by macrophages) and soluble antigens through the variable domain of B cell receptor (BCR), T cells exclusively detect antigens presented in the context of major histocompatibility complex (MHC) molecules through T cell receptors (TCRs) (Figure 1) [98].

Previous studies have indicated that B cells need complete native antigens in the early stage, especially in the form of immune complexes (with antibody and/or complement fragments), presented by macrophages located at the subcapsular sinus (SCS) of lymph nodes [55,99,100,101,102,103,104]. During this process, antigens are translocated by macrophages from the SCS lumen to the area underneath the SCS, where antigens are retained by macrophages, causing B cell accumulation under low motility and stimulating the activation of B cells, and then B cells internalize the antigens and move to the boundary between the T-B cell areas in lymph nodes [55,105,106]. The macrophages in the medulla are different from those in the SCS, but they all have been found to capture and retain antigen for up to 72 h after the initial antigen exposure [107]. Moreover, macrophage surface receptors, including CRs, PRRs, and carbohydrate-binding scavenger receptors, have been suggested to participate in the presentation of unprocessed antigens [108,109].

Macrophages are also able to present both endogenous and exogenous antigens to T cells as well as through the cross-presentation pathway, (Figure 1) [110,111,112]. The ability of macrophages to cross-present antigens to CD8 T cells could be important in the context of anti-tumor immunity. Several studies demonstrated that CD169^+^ macrophages contribute to cross-presentation either directly or by transferring antigens to CD8^+^ dendritic cells (DCs) in the spleen and sinus, which may facilitate the activation of CD8^+^ T cells [113] and invariant natural killer T (iNKT) cells [114] through MHC I or CD1d, respectively [115,116,117,118,119], while peritoneal macrophages may cross-present antigens to T cells directly [8,120,121]. In vivo studies show that F4/80^+^ CD169^+^ medullary sinus macrophages and F4/80^+^ CD169^−^ medullary cord macrophages have the ability of cross-presentation because only these cells can stimulate tumor-specific CD8^+^ T lymphocytes when targeted by a nanogel loaded with tumor-specific synthetic long peptide antigen (LPA) and a Toll-like receptor (TLR) 9 agonist [25]. However, considering the use of this highly artificial antigen, it is difficult to investigate the physiological relevance of these observations and it was not confirmed whether the macrophages processed the peptide directly on MHC-I for cross-presentation, or transferred it to DCs as mentioned for splenic and sinus CD169^+^ macrophages [122,123]. In the in vitro study, the tumor-infiltrating CD11b^+^ macrophages could efficiently cross-present to tumor-infiltrating CD8^+^ T lymphocytes but they were unable to provide costimulatory signals, including CD80, CD86, and intercellular Adhesion Molecule 1 (ICAM-1), which caused the loss of killing ability by the CD8^+^ T lymphocytes [124].

The pro- or anti-inflammatory phenotypes of macrophages could also impact their antigen presentation ability. It is suggested that pro-inflammatory macrophages might cross-present to reactivate effector CD8^+^ T lymphocytes when under sustained infections with the production of pro-inflammatory cytokines, such as interleukin (IL)-12 and IL-23 [8,125,126,127]. In contrast, cross-presentation by anti-inflammatory macrophages may have functions in immune tolerance against “self” proteins, food components and commensal microbes, similar to the cross-presentation of immature DCs [8,128]. A better understanding of macrophage antigen presentation abilities may help in the development of better adjuvants for cancer and infectious disease vaccines [104].

### 2.4. Different Phenotypes of Macrophages

Macrophages are capable of acquiring a spectrum of functional phenotypes that reflect the nature of the microenvironmental signals they receive. For simplicity, macrophages are often classified into two main phenotypes namely pro-inflammatory M1 (classically activated) and anti-inflammatory phenotype M2 (alternatively activated) that represent either end of the macrophage functional phenotype spectrum, (Figure 1) [129,130]. During inflammation, pro-inflammatory M1 macrophages play an important role in the host’s defense system, while anti-inflammatory M2 macrophages are related to vascular remodeling and tissue repair. Different macrophage phenotypes are involved in regulating the occurrence, development, and resolution of inflammation. However, when the polarization of M1 or M2 macrophages is dysregulated, it will almost always lead to chronic inflammation which may cause tissue damage and have a destructive effect on the human body [131].

#### 2.4.1. M1 Macrophages

**Figure 3:**
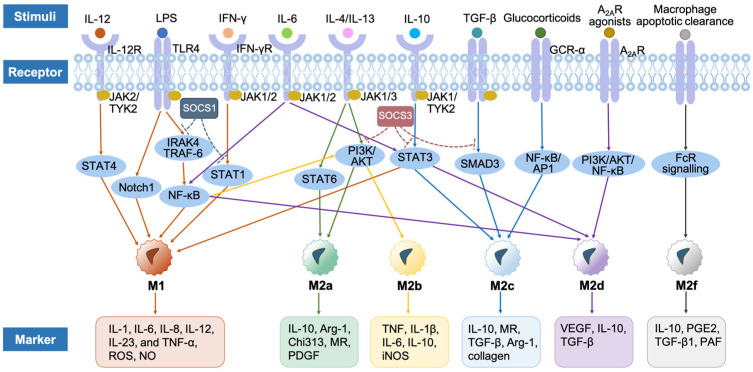
Signaling pathways of macrophage polarization. IL-12 activates M1 phenotype macrophages through the JAK/TYK2-STAT4 signaling pathway [144,145]. Monocyte-derived macrophages activate Notch1 and NF-κB under the stimulation of LPS and toll-like receptors, thus polarized M1 macrophages [146,147]. IFN works through the JAK-STAT1 signaling pathway to induce polarization of M1 macrophages [150]. IL-6 stimulates the M1 macrophages via the JAK-STAT3 signaling pathway [151]. SOCS1 can inhibit the signaling pathways of M1 polarization induced by NF-κB and STAT1 [149]. IL-4/IL-13 can lead to M2a phenotype via JAK-STAT6 and PI3K [152,153]. LPS and immune complexes can activate M2b macrophages through NF-κB signaling pathways [154]. IL-10 can drive the M2c activation by the JAK-STAT3 signaling pathway [11,155]. TGF-β activates the M2c phenotype through the SMAD3 signaling pathway [156]. Active glucocorticoids direct M2c polarization by binding to GCR-α, to interact with transcription factors including NF-κB and AP1 [32]. TLR signaling is an important trigger for NF-κB activation in the M2d activation [157]. IL-6 promotes M2d via the signaling of NF-kB and JAK/STAT3 pathways [158]. A2AR regulates the expression of M2d-associated chemokines and polarizing factors through PI3K/AKT/NF-κB pathways [159]. M2f macrophages are stimulated by macrophage apoptotic clearance related to phagocytosis of apoptotic cells, which is mediated by the FcR signaling pathway [160,161]. SOCS3 inhibits STAT3, PI3K, and SMAD3 [149]. In this figure, the solid arrow indicates the stimulation of the signaling pathways, and the dashed arrow indicates the inhibition of signaling pathways. Abbreviations: A2AR, adenosine A2A receptors; AKT, Ak strain transforming; AP1, activator protein 1; Arg-1, arginase-1; Chi3l3, chitinase3-like protein 3; FcR, Fc receptor; GCR-α, glucocorticoid receptor-α; IFN, interferon; IFN-γR, interferon-γ receptor; IL, interleukin; iNOS, inducible nitric oxide synthase; IRAK4, IL-1 receptor-associated kinase 4; JAK, Janus kinase; LPS, lipopolysaccharides; MR, mannose receptor; NF-κB, nuclear factor-κB; NO, nitric oxide; PAF, platelet-activating factor; PDGF, platelet-derived growth factor; PGE2, prostaglandin E2; PI3K, phosphoinositide 3-kinase; ROS, reactive oxygen species; SMAD, suppressor of mother against decapentaplegic; SOCS, suppressors of cytokine signaling; STAT, signal transducer and activator of transcription; TGF, transforming growth factor; TLR, Toll-like receptor; TNF, tumor necrosis factor; TRAF-6, TNF receptor-associated factor-6; TYK2, tyrosine kinase 2; VEGF, vascular endothelial growth factor.

M1 macrophages mainly secrete inflammatory cytokines and present antigens to T lymphocytes to initiate the adaptive immune response. following stimulation by DAMPs [38], PAMPs [22,132,133,134,135,136], and pro-inflammatory cytokines [137,138,139,140,141], as well as some growth factors [142], naïve macrophages are differentiated into the M1-like phenotype [143], (Table 1). While all these different stimuli support macrophage differentiation towards M1-like cells with an overall pro-inflammatory phenotype, they interact with different receptors and activate different signaling pathways. For example, IL-12 binds to IL-12 receptors (IL-12R) to promote the transphosphorylation of Janus kinase 2 (JAK2) and tyrosine kinase 2 (TYK2), which activates the translocation of signal transducer and activator of transcription 4 (STAT4) homodimer into the nucleus where they bind to STAT binding sites in the interferon (IFN)-γ promoter leading to M1 polarization [144,145]. Lipopolysaccharides (LPS) interact with TLR4 and downstream signaling molecules such as Toll/IL-1 receptor (TIR) domain-containing adaptor proteins, including MyD88 and TIR-domain-containing adapter-inducing interferon-β (TRIF) to activate a series of kinases including IL-1 receptor-associated kinase (IRAK)4, TNF receptor-associated factor (TRAF)-6, and IκB kinase (IKK), (Figure 3). These will then activate the nuclear factor-κB (NF-κB) and mitogen-activated protein kinase (MAPK) to regulate the expression of cytokines, chemokines, and type I IFNs [146,147]. On the other hand, IFN-γ initiates the JAK-STAT signaling pathway [148,149]. The signaling pathway of the TRIF adaptor can activate interferon-responsive factor 3 (IRF3), which in turn induces the secretion of IFN-α and IFN-β, and further activates the STAT [150]. Figure 3 provides an overview of the main signaling pathways involved in macrophage polarization.

M1 macrophages can secrete pro-inflammatory cytokines and mediators, such as IL-1, IL-6, IL-8, IL-12, IL-23, and tumor necrosis factor (TNF)-α, which mediate the production of iNOS and ROS, which participate in inflammation, damaged tissue sterilization and apoptotic cell removal and stimulate Th1 immune responses, (Table 1) [39,162,163,164]. Despite diverse stimuli and signaling pathways leading to the polarization of M1-like macrophages, the functional properties are more homogenous and encompass eliminating pathogens, clearance of debris and dead cells and killing tumor cells directly or indirectly by activating other immune cells. They can release IL-1 that induces the expression of anti-tumor cytotoxic lymphocytes from T lymphocytes, which directly kill tumors, or combine with activated lymphokine-activated killer (LAK) cells, natural killer (NK) cells, and tumor-infiltrating lymphocytes to kill tumors [57]. On the other hand, M1-like and M2-like tumor-associated macrophages (TAM) are also important components of the tumor microenvironment (TME). It is reported that the higher the ratio between M1-like and M2-like TAM, the longer the survival of cancer patients [165]. M1-like TAM can support chemotherapy in cancer by leading to a tumor immunological status to control tumor growth [166]. Therefore, reprogramming M2-like TAM to M1-like TAM is a promising therapeutic strategy to enhance anti-cancer immunity [167].

**Table 1:** Properties of macrophages.

	M1 (Classically Activated)	M2 (Alternatively Activated)				
**Subtype**	M1 [7,10,16,22,24,28,32,38,39,40,41,132,133,134,135,136,137,138,139,140,141,142,143,144,145,146,147,148,149,150,162,163,164,166,168,169,170,171,172,173,174,175,176,177,178,179,180,181,182,183,184,185,186]	M2a [7,10,16,32,152,153,174,175,187,188,189,190,191,192,193,194]	M2b [9,10,11,16,32,154,175,195,196,197,198]	M2c [9,10,11,16,18,32,155,156,174,175,194,199,200,201,202]	M2d [10,11,16,32,157,158,159,203,204]	M2f [16,160,161,205,206,207,208]
**Stimuli**	LPS, TNF, IFNs, TLR, TLR ligands, GM-CSF, IL-17A, IL-12, ANG-1	IL-4 (+M-CSF), IL-13	LPS, TLRs, IL-1R ligands, immune complexes	Glucocorticoids, IL-10, TGF-β	TLR, A2AR agonists, IL-6	Macrophage clearance of apoptotic cells
**Markers**	↑ROS, ↑TNF-α, ↑ IL-12, ↓IL-10	↑IL-10, ↑MR, ↓ROS, ↓TNF-α, ↓IL-12	↑IL-10, ↑MR, ↓ROS, ↓IL-12	↑IL-10, ↑MR, ↓ROS	↑IL-10, ↑VEGF, ↓TNF-α, ↓IL-12	↑TGF-β1, ↑MR
	IL-1β, IL-6, IL-8, IL-23, iNOS, CCL2, CD14, CD16, CD32, CD80, CD86, Calprotectin, MHC-II, PKM2, MARCO, PFKFB3, ACOD1	TGF-β, CCL17, CCL18, CCL22, CD36, CD163, CD301, IL-1Ra, Arg-1, IGF-1, MHC-II, CARKL, Ym1, Fizz-1, TREM2, IL1RN	IL-1β, IL-6, CCL1, CCL2, TNF-α, CD64, CD86, CD163, CCR8, VEGF, IGF-1, MHC-II, TNFSF14, PD-L1, SPHK-1	TGF-β, CD163, TLR1, TLR8, SLAM, SPHK-1, THBS1, HMOX-1	VEGF, MR, CD204, CD163, Arg-1, IDO, PGE2	IL-10, PGE2, PAF
**Signaling factors**	STAT1, STAT3, NF-κB (p65), IRF4, IRF5, Notch, AP-1, HIF1α	STAT6, SOCS1, PPARs, IRF4, GA TA3, KFL2, PI3K/AKT	STAT3, NF-κB (p50), IRF3, IRF4, Notch1, MAPKs, PI3K/AKT	STAT3, STAT6, NF-κB (p50), IRF4	STAT1, NF-κB (p50), IRF3	FcR pathway
**Functions**	Pro-inflammatory	Wound healing	Immunoregulatory	Immunosuppressive	Angiogenesis	Vessel morphogenesis
	Boost inflammation, sterilization, apoptotic cell removal, tumor killing, Th1 response	Anti-inflammatory, cell proliferation, cell migration, growth factors production, tissue remodeling, cell debris removal, Th2 response	Cell maturation, tissue stabilization, angiogenesis, ECM synthesis, tumor progression, tissue remodeling, Th2 response	Inflammatory resolution, tissue repair, ECM synthesis, growth factors production	Anti-inflammatory, tumor progression	Anti-inflammatory, cell differentiation, vessel stabilization and maturation

In this table, ↑ indicates increase, and ↓ indicates reduction.

#### 2.4.2. M2 Macrophages

M2 macrophages play an important role in tissue repair and are thought to stimulate angiogenesis and play a role in tissue remodeling. However, under pathological conditions, their unregulated activation leads to fibrosis and promotes tumor growth and invasion [209]. M2 macrophages can also be activated by a range of different stimuli including cytokines, TLR ligands, hormones, and growth factors [41,154,159,172,192,210,211,212,213,214]. Table 1 provides a summary of different stimuli that support M2-like polarization. The resulting M2-like cells seem to have a more heterogeneous functional spectrum based on the nature of stimuli and can be divided into different subtypes including M2a, M2b, M2c, M2d, and M2f with distinct molecular signatures, cytokine profiles and functions as highlighted in Table 1. It is worth noting that the majority of these classifications are based on in vitro conditions and in vivo conditions may present an even more complex and dynamic picture.

Like M1 cells, there are different signaling pathways involved in mediating the effect of pro-M2 stimuli, (Figure 3). For example, IL-4 and IL-13 secreted by innate and adaptive immune cells bind to JAK1 and JAK3 through IL-4 receptor α (IL-4Rα), which then triggers STAT6 and activates the M2a macrophages. M2a can also be activated through the phosphoinositide 3-kinase (PI3K)/Ak strain transforming (AKT) signaling pathway induced by IL-4 [152,153]. M2a macrophages secrete IL-10, arginase-1 (Arg-1), chitinase3-like protein 3 (Chi3l3), MR, and platelet-derived growth factor (PDGF) [189]. They play an essential role in wound healing because they can release anti-inflammatory mediators and growth factors to support cell proliferation and migration, as well as tissue remodeling [7]. Furthermore, they can also stimulate the recruitment of basophils, eosinophils, and Th2 cells to remove cell debris together [190,191].

LPS, TLR, IL-1R ligands, and immune complexes can activate M2b macrophages through NF-κB (p50) signaling pathways [154]. In the meantime, NF-κB (p50) and IRF3 play a core role in activated lymphocyte-derived DNA (ALD-DNA)-induced M2b polarization, and here, the translocation of NF-κB (p50) into the nucleus is mediated by PI3K and MAPK pathways [154]. M2b macrophages express TNF, IL-1β, IL-6, IL-10, iNOS, and low levels of IL-12 [196], which is involved in the regulation of immune responses, including the modulation of cell maturation, tissue stabilization, angiogenesis, extracellular matrix (ECM) synthesis, and tumor progression, as well as the recruitment of eosinophils and Th2 cells [11,154,197,198].

IL-10, glucocorticoids, and transforming growth factor (TGF)-β induce the polarization of M2c macrophages [32]. IL-10 polarizes the M2c phenotype through the induction of p50 NF-kB homodimer and STAT3 activities under the stimulation of IL-10 receptors, including IL-10Ra and IL-10Rb, which belong to the interferon receptor (IFNR) family [11,155]. Active glucocorticoids bind to the glucocorticoid receptor (GCR)-α, to interact with transcription factors including NF-κB and activator protein 1 (AP1), which further directs M2c polarization [32]. TGF-β binds to the TGF-β receptors (TβRs) and the downstream mediators of TGF-β signaling are suppressor of mother against decapentaplegic (SMAD)-dependent pathways to activate the M2c phenotype [156]. M2c macrophages express IL-10, TGF-β, MR, and Arg-1, and promote the production of growth factors, polyamines, and collagen, which are beneficial to tissue repair and regeneration [18,201,202].

M2d macrophages, also known as M2-like TAM, are polarized by TLR, adenosine A_2A_ receptors (A_2A_R) agonists [203], and IL-6. TLR and IL-1R signaling are important triggers for NF-κB activation in the TAM activation [157], while A_2A_R regulates the expression of TAM-associated chemokines and polarizing factors through PI3K/AKT/NF-κB pathways [159], and IL-6 promotes TAM by binding to its two distinct receptors, including IL-6R and gp130, to trigger signaling of NF-kB and JAK/STAT3 pathways [158]. TAM secretes vascular endothelial growth factor (VEGF), TGF-β, and IL-10, and low levels of TNF-α, IL-12, and IL-1β to induce tumor blood vessel growth and angiogenesis [204].

M2f macrophages are stimulated by macrophage clearance of apoptotic cells related to phagocytosis of apoptotic cells. They can up-regulate the expression of MR and anti-inflammatory modulators, including TGF-β1, IL-10, prostaglandin E2 (PGE2), and platelet-activating factor (PAF), which is mediated by the FcR signaling pathway [160,161]. In the meantime, they facilitate pericyte cell and smooth muscle cell differentiation and promote endothelial cell migration to process vessel stabilization and maturation [161,205,206]. In addition to the above cytokines and receptor proteins, hypoxia-inducible factor (HIF) and transcription factors, such as peroxisome proliferator-activated receptor γ (PPARγ) and Krueppel-like factor 4 (KLF-4) also promote M2 phenotype [207,208].

#### 2.4.3. Transitions between M1 and M2

**Figure 4:**
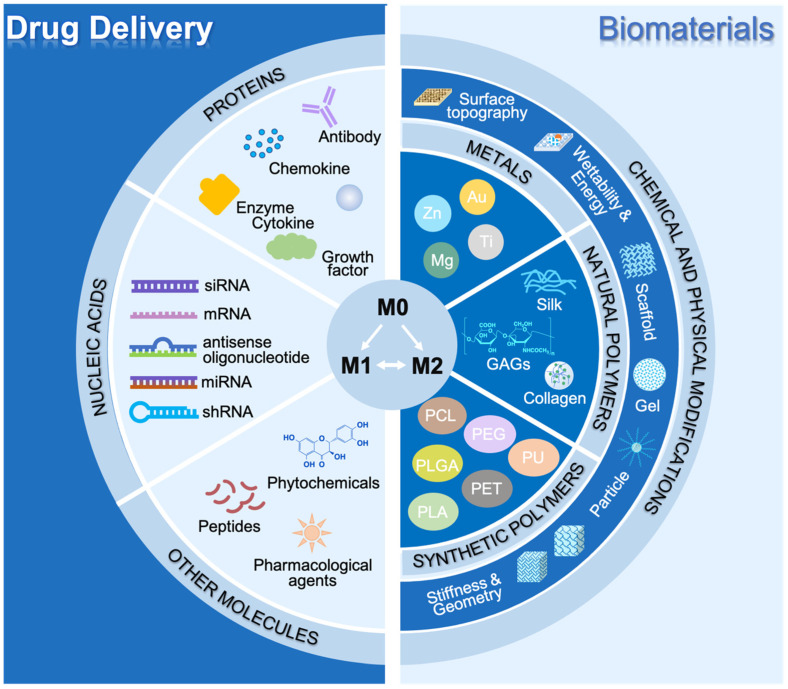
Therapeutic strategies for modulating macrophage polarization. (1) Drug delivery: immune-instructive modulators, including proteins (such as cytokines, chemokines, antibodies, growth factors, and enzymes), nucleic acids, as well as other anti-inflammatory and/or pro-wound-healing molecules are delivered to damaged tissue, aiming to intervene at different pathway points for the reprogramming of macrophages polarization [173]; (2) Biomaterials: Biomaterials, including metallic materials, natural polymers and synthetic polymers are applied to regulate macrophage polarization to affect the biological behavior of immune factors, while their chemical and physical properties can also affect macrophage phenotypes [30,215,217]. Abbreviations: GAGs, glycosaminoglycans; miRNA, microRNA; mRNA, messenger RNA; PCL, polycaprolactone; PEG, polyethylene glycol; PET, polyethene terephthalate; PLA, polylactic acid; PLGA, poly(lactic-co-glycolic acid); PU, polyurethane; shRNA, small hairpin RNA; siRNA, small interfering RNA.

In a normal and uncomplicated wound healing response following injury, there is a gradual transition between the initial pro-inflammatory responses driven by M1-like cells towards pro-healing M2-driven responses [7]. While the initial acute inflammation triggers clearance of dead cells, pathogens and other harmful factors, the pursuant pro-healing responses set the scene for repair, vascularization and remodeling of tissue to reinstate tissue homeostasis [3,7,18]. The transition between different macrophage phenotypes stems from their plasticity and ability to respond to changes in the signals they receive from their microenvironment [7,18,215]. Many pathologies (e.g., presence of a foreign body, diabetes, cancer, inflammatory and vascular diseases) could disturb such transition, leading to chronic inflammation and tissue damage [24]. The realization of macrophage plasticity has motivated extensive research into its understanding and also leveraging it therapeutically including the development of ‘immune-instructive’ niches to modulate macrophage functional phenotypes to reinstate tissue homeostasis, promote healing or even boost immune responses against pathogens or tumor cells [173,215,216,217,218,219,220,221]. In the following sections, we will provide an overview of different strategies for modulating macrophage phenotype, (Figure 4).

## 3. Drug Delivery for Modulating Macrophage Polarization Therapeutically

There has been a longstanding interest in using biomolecules such as proteins (e.g., cytokines, chemokines, antibodies, growth factors and enzymes), nucleic acids and small molecules for modulating macrophage phenotypes with some of these approaches being translated to the clinic [173]. The delivery methods for these molecules are diverse, being adapted to various clinical requirements, and include nanoparticles, viral vectors, liposomes, microspheres, hydrogels, scaffolds, and oligopeptide complexes. The ultimate aim in all these scenarios is to target specific signaling pathways for reprogramming of macrophage functional phenotype [173].

### 3.1. Proteins

Cytokines, growth factors, and enzymes actively regulate macrophage polarization [7,10,39]. Meanwhile, chemokines and their receptors are able to affect the phenotype of macrophages by directly and indirectly promoting cellular infiltration and recruitment after tissue injury [7,24,143]. Thus, these immune players are promising molecules for sustained release from bioactive delivery systems to control the M1-M2 balance for the treatment of various diseases, such as inflammatory diseases, including atherosclerosis, arthritis, diabetes, sepsis, bowel disease, angiogenesis, autoimmune diseases, such as autoimmune neuropathies and myocarditis, and cancer, as well as promoting damaged tissue healing [10,18,24,38,41,143,166].

Cytokines can be directly delivered or produced via macrophages and other immune cells already present in a damaged area. Among the various cytokines for M2 macrophage induction, manipulation of the local concentrations of IL-4, has been widely used to stimulate the M2a phenotype to reduce inflammatory conditions and facilitate tissue regeneration [7,187,188,189]. For instance, Raimondo et al. [188] produced IL-4-conjugated gold nanoparticles and injected them into the injured skeletal muscle in the murine model, which showed a twofold increase in the percentage of M2a macrophages and an approximately twofold decrease in M1 macrophages, leading to the improvement in histology with around 40% increase in muscle force, compared with mice treated with vehicle only. This indicated that M2 macrophages are essential for the regeneration of functional muscle fibers, which can promote the differentiation of myogenic precursor cells and the formation of mature myotubes [222]. TGF-β is a growth factor that plays an essential role in M2 macrophage polarization [212,223]. The TGF-β1-loaded thermosensitive photocrosslinkable glycidyl methacrylate-modified-hydroxypropyl chitin hydrogel (GM-HPCH) has been developed to shift recruited macrophages from M1 to M2 and stimulate chondrogenic gene expression to promote chondrogenesis in the rat chondral defect region, which further stimulates the migration of marrow stromal cells and promotes cartilage healing within 12 weeks [224].

Additionally, the application of anti-TNF-α antibodies has been recognized as an immune-modulatory strategy to block the inflammatory effect of TNF-α for attenuating inflammation during tissue repair [173]. Many different therapeutic anti-TNF-α antibody formulations are commercially available, including monoclonal antibodies (mAbs), such as adalimumab, infliximab, and golimumab, antibody fragments, such as certolizumab pegol, and fusion recombinant proteins, such as Etanercept, using as therapeutic methods for many autoimmune diseases such as diabetes, as well as promoting wound healing [43,173]. For example, the topical addition of anti-TNF-α neutralizing antibodies shifted the macrophage phenotype towards M2 and accelerated wound healing in the in vivo study [43]. In another study, Wang et al. designed a glucose-sensitive system consisting of a chitosan and collagen scaffold to deliver anti-TNF-α antibodies, which was shown to attenuate the inflammatory response and promote alveolar bone healing in a diabetic rat model with alveolar bone defects by inhibiting the NF-κB signaling pathway to reduce the expression of local TNF-α and inflammatory factors, such as chemokine (C-C motif) ligand (CCL)2 and C-X-C motif chemokine ligand (CXCL)1, and increase the production of the osteogenesis-related proteins, including alkaline phosphatase (ALP), type I collagen, osteocalcin, runt-related transcription factor 2 (Runx2), osterix, and bone morphogenetic protein 2 (BMP2) [225].

Stimulating the expression of pro-inflammatory regulators or preventing the production of anti-inflammatory mediators in the tumor environment to transit TAM cells from M2 to the M1 phenotype are important strategies for cancer therapy [21,22,143,166,204]. The study conducted by Liu et al. described the loading of nanocomplex Catalase-Ce6 with immobilized hydrophilic catalase protein in the M1 macrophage extracellular vesicles (EVs), which was subsequently assessed on the subcutaneous mouse forestomach carcinoma cell line tumor model. The results revealed that it could effectively enhance the ratio of ROS from photodynamic therapy (PDT) and successfully repolarize M2 macrophage to M1 type in the tumor tissue, thus improving the efficacy of PDT [226]. Guiducci et al. applied adenoviral delivery of the CCL16 chemokine, TLR 9 ligand CpG and anti-IL-10 receptor antibody to promote the recruitment of macrophages and DCs at the site of pre-established tumor nodules. These experiments showed a transition of infiltrating macrophages from M2 to M1 to produce more TNF and IL-12 and triggered innate response debulking large tumors within 16 h, which indicated that innate resistance mechanisms and proinflammatory cytokines like TNF play a central role in tumor destruction [227].

Targeting macrophage polarization with protein based drugs has emerged as a popular strategy to for the treatment of inflammatory diseases and cancer immunotherapy, among which, the most commonly reported proteins are antibody antagonists with high selectivity of action that target cell-surface receptors or extracellular chemokines and cytokines [173,228,229]. Some protein drugs have been approved for clinical trials or application, including the IL-6-antibody, CD47-antibody, natalizumab, and canakinumab [228]. But because of the timescales of tissue restoration and the low stability of proteins, frequent administrations of these drugs may cause safety concerns and possibly limit translational potential [228]. Therefore, the targeted protein drug delivery system is required to improve the performance of macrophage behavior modulation in preclinical studies by enabling their controlled release at diseased sites [173,230]. However, developing naturally occurring protein delivery systems with significant therapeutic effects requires a long-term process and a large amount of funding investment [231]. In the meantime, the use of biomaterials that are foreign to the body, such as polymers, lipids, or hydrogels and protein aggregation caused by the delivery system may elicit immune responses and potential adverse reactions [231]. To accelerate clinical translation, the physicochemical properties of the drug delivery systems should be optimized and the manufacturing processes need to be adjusted to minimize or completely remove the harmful components related to depot preparation to increase the shelf-life and stability of the proteins and achieve the controlled release of these drugs after the agents are administered [228,229].

### 3.2. Nucleic Acids

Nucleic acid therapy was initially developed to insert designed genes as DNA duplexes into the deficient cells to achieve the desired function [24]. With the development of molecular biology methods, synthetic biology and bioengineering, the nucleic acid platforms have been extended to several types for therapeutic applications, including messenger RNA (mRNA), antisense oligonucleotides, small interfering RNA (siRNA), microRNA (miRNA), and small hairpin RNA (shRNA) [27]. The biggest challenge for nucleic acid therapy is to maintain the bioavailability of DNA or RNA strands when encountering physiological barriers because naked unprotected nucleic acids would be degraded rapidly by extra and intracellular nucleases in the biological fluids. Further, because of their high molecular weight, polar nature, and poor permeation across the nuclear membrane, it is more difficult for nucleic acid to translocate to the nucleus where the molecular machinery is present to achieve translation [27]. As a result, the delivery systems are required to preserve nucleic acid structural integrity, promote cellular uptake, endosomal release, and nuclear penetration, and avoid immunological reactions.

Commonly used gene delivery systems include viral vectors, such as adenoviruses, retroviruses, and lentiviruses, and non-viral vectors, such as lipoplexes, polyplexes, polymer systems, and hybrids, among which the non-viral vectors have gained more popularity due to the simplicity of approach and lack of possible viral pathogenicity [27]. There are three main delivery strategies for internalization of nucleic acids in cells: (1) release of genes loaded in particles after being engulfed in the phagosome and then breakdown in the acidic lysosomal compartment; (2) cytoplasmic delivery via endocytosis without phagosome formation or lysosome fusion; (3) active transport into cells through coat proteins, such as clathrin and caveolin [27]. Here, we focus on gene delivery for the regulation of macrophage-related functions to alleviate inflammation, promote healing or boost immune responses against tumor cells.

Virus-mediated gene delivery is a method to inject DNA duplexes inside host cells and achieve the replication of their genes with high efficiency by harnessing the ability of viruses to protect transgenes from degradation and effectively infect various cell types [27]. The delivery of genes encoding specific molecules by viral vectors has been used to modulate macrophage phenotypes for immunotherapies [24]. Tabata et al. found that macrophage transfection with the Glipr1 gene using an adenoviral vector induced phosphorylation of JNK, p38, and ERK to activate MAPK-signaling pathways, which increased the production of CD40, CD80, MHC class II molecules, IL-6 and IL-12 in vitro and reduced prostate tumor growth and lung cancer metastasis in vivo [232]. Similarly, an approach was adopted for macrophage transfection with the IL-10 encoding lentivirus to direct macrophage polarization towards an anti-inflammatory phenotype (M2) even in a pro-inflammatory environment with inflammatory stimuli (LPS) addition, and it was demonstrated to reduce TNF-α production through suppressing NF-κB activation [233].

However, viral vectors are more likely to induce immune responses by the host and there are also concerns about their carcinogenic potential, so novel non-viral substitutes with high biocompatibility have been investigated to improve the pharmacokinetics and pharmacodynamics of nucleic acid delivery with minimal adverse side-effects [27,234,235,236,237,238,239,240]. Although the efficiency of this system in gene transduction is not as high as that of viral systems, nonviral delivery systems have better cost-effectiveness and availability, as well as lower limitations in the size of transgenes, compared to viral systems [241,242]. The significant advances in nanotechnology and bio-material science have led to the development of various biocompatible and biodegradable materials for nucleic acid encapsulation and delivery. Nanoparticles (1~1000 nm) have been widely used as non-viral delivery strategies for macrophage therapeutics and, in particular, lipid-based nanoparticles have been extensively studied [243]. Li et al. proposed that lipidoid nanoparticle-mediated delivery of IRF5 siRNA into the macrophages infiltrated in the wound of spinal cord injury mice facilitated the M1 to M2 transition, reduced demyelination and neurofilament loss, and promoted functional recovery, by controlling the expression of pro-inflammatory cytokines (TNF-α and IL-1β) downstream of MyD88-dependent TLR signaling [244]. In other studies for cancer immunotherapy, a pH-sensitive cationic lipid nanoparticle was used to deliver siRNA to TAMs and achieve anti-tumor therapeutic response by silencing the STAT3 and HIF-1α, which increased infiltrated macrophages (CD11b^+/−^ cells) in the TME as well as the density of M1 macrophages (CD169^+/−^ cells), thus resulting in reversing the pro-tumorous functions of TAMs-mainly angiogenesis and tumor cell activation [245].

The modification of nanoparticle surfaces with proteins and peptides can improve their specific cell targeting due to receptor-mediated specificity [243]. IL-10 encoding plasmid DNA was encapsulated into non-condensing alginate-based nanoparticles with surface modification of tuftsin peptide by Jain et al. to achieve active macrophage targeting. In a rat model of arthritis, this treatment successfully increased the percentage of M2 macrophages (66%) upon intraperitoneal administration, compared with the untreated group, and significantly reduced the production of pro-inflammatory cytokines (TNF-α, IL-1β, and IL-6) expression in the joint tissue, showing the prevention of the inflammation and joint damage as revealed by magnetic resonance imaging and histology [42].

Lipid-based delivery systems, especially EVs, are esteemed as promising delivery vehicles for various genetic therapeutics because they are relatively inert, non-immunogenic, biocompatible and biodegradable [246]. Liu et al. developed immunoregulatory EVs by decorating M1-macrophage-derived EVs with vesicular stomatitis virus glycoprotein (VSV-G), a pH-responsive viral fusion protein, and electroporating anti-PD-L1 siRNA (siPD-L1) into the EVs. After the administration of this virus-mimic nucleic acid-engineered EVs to the CT26 tumor-bearing mice, the fusion of VSV-G with cells promoted the release of siPD-L1 into the cytoplasm and triggered robust gene silencing, causing the efficient block of PD-L1/PD-1 interaction and the secretion of IFN-γ produced by CD8^+^ T cells, which stimulated the repolarization of M2 TAM to M1 macrophages [247].

The delivery of nucleic acids is a promising approach for finely and specifically regulating the transition between M1 and M2 macrophages [27]. Gene therapy has been exploited to increase the secretion of mediators related to the M2 phenotype to control inflammation, whereas antisense therapy has been applied to selectively reduce the expression of targeting molecules to decrease either M1-associated regulators in inflammatory conditions or M2-associated modulators in the TME [173]. The development of novel nano-delivery systems and their specific targeting modification have led to dramatic advances in controlling macrophage polarization in different pathologies [243]. However, this approach still faces important challenges, which are mainly about safety concerns and the stability of these delivery systems in in vivo settings [27,173,234,235,236,239]. Therefore, more fundamental and translational research is desired to address these problems for future clinical application.

### 3.3. Other Molecules

Peptides are small molecules, and they are less expensive to manufacture compared with full-length proteins because of their small chains of amino acids and simple structure. Some anti-inflammatory peptides, such as α-melanocyte-stimulating hormone (α-MSH) [248,249], microglial healing peptide 1 (MHP1) [250], suppressors of cytokine signaling (SOCS)1-KIR [251], and chromofungin [252] have been utilized to control inflammatory diseases and tissue injury, through modulating macrophage pro-inflammatory functions. Gunassekaran et al. harnessed M1 macrophage-derived exosomes transfected with NF-KB p50 siRNA and miR-511-3p and surface-modified with IL-4RPep-1, an IL-4R-binding peptide, named IL4R-Exo(si/mi), to foster M1 polarization and target IL4R for the inhibition of tumor growth by reprogramming TAMs into M1-like macrophages in mice inoculated with breast or lung tumor cells. Results showed that IL4R-Exo(si/mi) successfully decreased the levels of M2 markers and cytokines, including Arg-1, TFG-β, IL-10, and IL-4 and increased the expression of M1 cytokines, including IL-12 and IFN-γ in the lymphomononculear population of tumor tissues [253]. There are also examples of modulating macrophage phenotypes using peptide fragments that engage specific receptors on macrophages. Cha et al. showed that integrin α2β1 peptide (the type I collagen α1(I)-CB3 fragment Asp-Gly-Glu-Ala) coated surfaces promote macrophage polarization towards an M2 phenotype as evidenced by a significant increase in CD206 and IL-10 expression by macrophages after 6 days of culture [254].

In addition to peptides, some soluble pharmacological anti-inflammatory agents such as dexamethasone, heparin, and melatonin have been encapsulated in delivery systems for the treatment of inflammation [30]. Due to the complex pharmacokinetics of these agents and reduced drug bioactivity, their use for long-term effects has been limited. Nevertheless, their use in combination with sustained-release delivery systems could offer therapeutic opportunities. For example, Lee et al. proposed using thiolate PLGA nanofibers conjugated with mono-(6-mercapto-6-deoxy)-β-cyclodextrin (SH-β-CD) containing dexamethasone (DEX) and ropivacaine (RVC) for the treatment of neuropathic pain in rats with the injured sciatic nerve, which restricted drug flow to the motor nerve and reduced the expression of TRPV1 which is involved in the signal transduction of nociceptors and detects noxious heat and pain. Results revealed that PLGA-CD-DEX-RVC nanofibers showed long-term anti-inflammatory effects and promoted M2 macrophage polarization consistently leading to the relief of allodynia cold sensitivity for up to 14 days [255].

Furthermore, phytochemicals isolated from plants and microbes as modulators of M1-M2 macrophages have been discussed in a previous review [45]. Some of them have been proposed to design or combine with nanocarriers to regulate immune responses by interacting with macrophage polarization [23,173,256]. The spectrum of polarizing macrophage modulators that are potentially applied in tissue engineering, ranges from proteins to small molecules, such as chemical compounds [173]. The release of pro-/anti-inflammatory agents from particles or scaffolds, as well as other delivery systems, is an effective strategy for inflammatory diseases and cancer therapy. However, more studies are required to fully assess the clinical potential of these agents.

## 4. Advanced Biomaterials for Macrophage Polarization Therapeutically

### 4.1. Biomaterials

In addition to modulating macrophage behavior using drugs and biomolecules, it has also been well established that macrophages respond to the physio-chemical properties of materials, such that a biomaterial itself can be designed to regulate macrophage polarization, affect different biological processes and ultimately resolve clinical problems [26,30,205,215,220,257,258,259]. Biomaterials can be classified into metallic materials, such as stainless steels, titanium, and their alloys, and non-metallic materials, including natural polymers, such as glycosaminoglycans (GAGs) and collagen, and synthetic polymers, such as PLGA, polycaprolactone (PCL), and polytetrafluoroethylene (PTFE), (Figure 4) [215,220,258,260,261,262,263]. The underpinning biological-material interactions that govern how macrophages respond to biomaterials are complex and the main aspects remain poorly understood, however, significant advances have demonstrated how surface chemistry, topography, wettability, geometry, and material mechanics can be altered to predictably modulate macrophage behavior and, ultimately, the immune response.

#### 4.1.1. Metallic Materials

Since steel was created for implants in the early 1900s, metallic materials such as titanium, gold, niobium, tantalum, and their alloys, as well as stainless steels, have been developed for use in biomedical applications [263,264,265]. Most of these metallic biomaterials are broadly used for hard tissue replacement, such as bone repair in oral, maxillofacial, and craniofacial surgeries [264,265]. Titanium is used extensively due to its remarkable mechanical properties and biocompatibility. It also demonstrates a very strong apatite-forming ability following implantation, which is thought to be a crucial component in osteoinduction [264].

Recently, biodegradable metals (BMs), including magnesium, iron, zinc, and their alloys, have been applied as temporary support during the healing process, which thereafter can be degraded through electrochemical corrosion with body fluid [263,264,265]. The most effectively utilized in clinical settings are magnesium-based BMs because of their osteopromotive quality, adaptable biodegradability, and outstanding biocompatibility [263,264]. Research findings reveal that in the initial stages of inflammation Mg^2+^ enhanced the recruitment of monocytes and promoted their differentiation into macrophages that release cytokines for bone healing whilst, in the subsequent stages of bone regeneration, it persistently triggered the NF-κB signaling pathway in macrophages leading to an increase in osteoclastic-like cells and a slowdown in bone maturation [266,267].

Metal-containing bioactive nanomaterials, including metal-organic framework, metal sulfide, metal oxide, and metal carbide, have garnered a lot of interest in therapeutically delivering drugs targeting macrophage phenotypes for inflammatory disease treatment and cancer therapy as they have good antibacterial and antioxidant properties in addition to photocatalytic and magnetic properties [258,268]. According to certain in vitro research, macrophages can be redirected to the M2 profile by using zinc oxide or copper and iron oxide nanoparticles, which lowers the release of pro-inflammatory cytokines [269,270]. Other in vivo investigations demonstrated that zinc, titanium, and cerium oxide nanoparticles markedly reduced acute inflammation in burn injuries, pneumonia, and autoimmune diseases [271,272,273,274].

Metallic biomaterials are currently the most widely used materials for medical devices that replace tissue (bone) [264]. However, interactions between macrophages and metal materials are regulated by a variety of factors including the size, mechanics, surface, chemistry, and topography, as well as the geometry of the metals [265,275]. Therefore, for clinical use, appropriate material selection, meticulous design, and surface modification are important. These aspects will be discussed in the chemical and physical modification section.

#### 4.1.2. Natural Polymers

**Figure 5:**
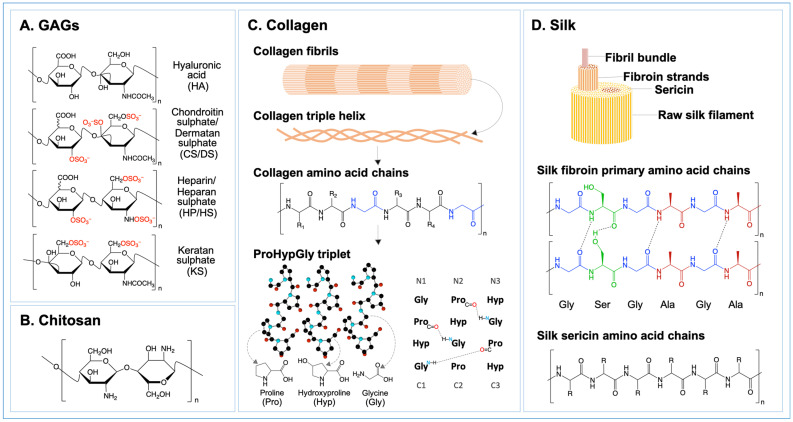
Chemical structures of GAGs (**A**), chitosan (**B**), collagen (**C**), and silk (**D**). (**A**) GAGs: GAGs are divided into HA, CS/DS, HP/HS, and KS, based on different disaccharides pairs in their chains; (**B**) Chitosan: chitosan is a β-(1,4) glycosidic linkage-linked linear polysaccharide consisting of D-glucosamine and N-acetyl-D-glucosamine units; (**C**) Collagen: collagen three polypeptide chains wrap around each other to form a left-handed polyproline II-type helix to create a right-handed triple helix maintained mainly by interstrand hydrogen bonds, which mandates that every third residue be Gly, and ProHypGly is the most common triplet in collagen triple helix; (**D**) Silk: two proteins make up the silk fibroin skeleton: fibroin, which is the inner layer protein, and sericin, which is the outer-layer covering protein [279,280,281,282]. Abbreviations: CS/DS, chondroitin sulphate/dermatan sulphate; GAGs, glycosaminoglycans; Gly, glycine; HA, hyaluronic acid; HP/HS, heparin/heparan sulphate; Hyp, hydroxyproline; KS, keratan sulphate; Pro, proline.

Natural polymers are biological materials sourced from animals or plants that elicit a distinct host response because of their diverse physical and chemical characteristics, such as surface topologies and ligand landscapes [30,259,276]. Among them, ECM derived materials have been extensively researched and found to impact the behavior of immune cells during tissue remodeling [30,215,277]. For instance, in preclinical animal investigations, scaffolds made from urinary bladder matrix and small intestinal submucosa were shown to enhance the localized M2:M1 ratio around bioscaffolds and induce a positive, functional tissue remodeling response [278]. Other compositions of natural polymers that are similar to ECM, including polysaccharides like glycosaminoglycans (GAGs) and chitosan or proteins like collagen and silk, (Figure 5), are well-developed and applied to build highly biocompatible scaffolds to control macrophage polarization with several examples of their use in regenerative medicine and drug delivery applications, owing to their favorable properties such as bioactivity, biocompatibility, and biodegradability [220,259,277,279,280,281,282].

**Figure 6:**
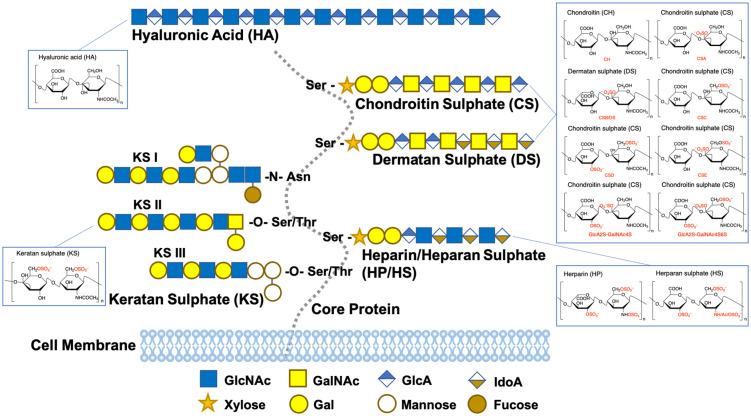
Chemical structures of GAGs. HA: β-(1,4)-GlcA and β-(1,3)-GlcNAc. CS: β-(1,4)-GlcA and β-(1,3)-GalNAc. DS (CSB): α-(1,4)-IdoA and β-(1,3)-GalNAc. CH: non-sulphated GlcA-GalNAc; CSA: GlcA-GalNAc4S; CSC: GlcA-GalNAc6S; CSD: GlcA2S-GalNAc6S; CSE: GlcA-GalNAc4S6S; chondroitin 2,4-sulphate: GlcA2S-GalNAc4S; chondroitin 2,4,6-sulphate: GlcA2S-GalNAc4S6S. HP/HS: β-(1,4)-GlcA/α-(1,4)-IdoA and α-(1,4)-GlcNAc. KS: β-(1,3)-Gal and β-(1,4)-GlcNAc. HA is not covalently linked to the proteoglycan but HP/HS and CS/DS are assembled to a proteoglycan via a serine residue, and the KS chain can be N-linked to an asparagine residue (KS I) or O-linked to a serine or threonine residue (KS II and KS III) on a proteoglycan [297,298,299]. Abbreviations: Asn, asparagine; CS/DS, chondroitin sulphate/dermatan sulphate; GAGs, glycosaminoglycans; Gal, galactose; GalNAc, N-acetylgalactosamine; GlcA, glucuronic acid; GlcNAc, N-acetylglucosamine; HA, hyaluronic acid; HP/HS, heparin/heparan sulphate; IdoA, iduronic acid; KS, keratan sulphate; Ser, serine; Thr, threonine.

GAGs are linear polysaccharides, including hyaluronic acid (HA), chondroitin sulphate/dermatan sulphate (CS/DS), heparin/heparan sulphate (HP/HS), and keratan sulphate (KS), divided by different disaccharides pairs in their chains, (Figure 6) [282,283]. As an important part of ECM, they participate in extra-/intra-cellular signaling and secretion of cytokines and growth factors, (Table 2) [282,283]. HA has both pro- and anti-inflammatory properties, determined by its molecular weight. High molecular weight HA (HMW-HA) (>500 kDa) promotes regression of inflammation, while low molecular weight HA (LMW-HA) 20~250 kDa) is beneficial to the progression of inflammation and wound healing [284,285,286]. Chemical modifications of HA have been investigated to improve biofunctionality, with a focus on the addition of sulphate groups to create HS-like biomolecules [287,288,289,290,291,292,293]. Chemically sulphated HA is reported to have a more potent anti-inflammatory effect when compared to plain HA, whereby the pro-inflammatory characteristics exerted by M1 macrophages were reduced and the levels of the pro-inflammatory cytokines were decreased [277]. It is reported that sulphated HA interrupts inflammasome activation on the level of TLR-mediated transcriptional priming of inflammasome components and downstream effector molecules, such as preventing phosphorylation of NF-κB and consequent transcription of NF-κB controlled pro-inflammatory genes [294,295,296].A hydrogel incorporated with sulphated HA was developed to deliver sulphated HA to diabetic mice wounds over a period of at least one week, which enhanced the activation of pro-regenerative macrophages, reduced inflammation, promoted vascularization, and accelerated the formation of new tissue and wound healing [296].

The main effect of CS/DS on macrophages is promoting the anti-inflammatory M2 phenotype by regulating the NF-κB and TLR signaling pathways [334]. Because CS/DS is readily soluble in water, the process of producing CS-based biomaterials to aid in wound healing typically involves crosslinking or binding with other polymers such as HA, gelatin, and chitosan [220,334,335]. For example, oxidized chondroitin sulphate and hydroxybutyl chitosan were covalently crosslinked to make hydrogels, which reduced the in vitro and in vivo pro-inflammatory gene expression of macrophages, including IL-1β and TNF-α, and decreased lymphocyte and macrophage infiltration around the implanted hydrogel after 7 days treatment on a murine subcutaneous implantation model [336]. HP/HS coordinates different levels of inflammation by interacting with numerous molecules expressed on the cell surface, such as selectins and integrins, activating cytokines and chemokines, such as the interleukin family, and inhibiting the pro-inflammatory enzymes and cytotoxic mediators, such as elastase, eosinophil peroxidase, and stromal-derived factor-1 [319,320,321,322,323,324,325,326]. Although some studies showed that they can promote M1 macrophages [327,328], they are well known for their capabilities to bind and enhance the functions of pro-angiogenic growth factors, which can be used to promote wound healing [337,338,339,340,341]. For example, heparin and konjac glucomannan were co-polymerized to form an injectable hydrogel system, in which, heparin was applied as functional moieties to sequester the macrophage-produced GFs by binding numerous pro-angiogenic GFs. In the in vivo study with a mice wound healing model, results showed that abundant and mature blood vessels are found in the gels after 14 days of subcutaneous implantation, which is indicated by the high expression of α-smooth muscle actin (SMA) and CD31 [340].

Structurally similar to GAGs, chitosan is also well-developed in tissue engineering applications. Chitosan is a β-(1–4) glycosidic linkage-linked linear polysaccharide consisting of D-glucosamine and N-acetyl-D-glucosamine units, (Figure 5B) [342]. Chitosan is derived from chitin, which has favorable biodegradability, biocompatibility, immune-modulatory and gel-forming properties [262,343]. Chitosan is reported to promote macrophages into anti-inflammatory phenotypes [343,344,345]. However, it is worth noting that due to its poor solubility in neutral and basic media, chitosan is more frequently modified or combined with another polymer to make hydrogels than used in hydrogels made solely of chitosan. Collagen has a natural network-like structure making it suitable to form highly organized 3D scaffolds, (Figure 5C) [261]. It is also biocompatible and is able to provide biochemical cues for cell adhesion, proliferation, and differentiation, but collagen hydrogels are typically difficult to functionalize so cannot be readily tuned to provide a specific cell response, and have poor mechanical characteristics [342]. Compared with collagen, chitosan is much more versatile in terms of structure and chemistry [342]. These properties have made the combination of collagen and chitosan an attractive choice for fabricating 3D scaffolds and hydrogels to support tissue regeneration while modulating inflammatory responses [257,342,346]. For example, the sulphated chitosan-doped collagen type 1 hydrogel was demonstrated to reduce M1 polarization and pro-inflammatory cytokines but increased anti-inflammatory molecules and facilitated the trans-differentiation of macrophages into fibroblasts. This resulted in the formation of collagen and ECM and increased angiogenesis, which are both beneficial in the resolution of wound healing [346]. You et al. carried out several studies and demonstrated the use of nanosilver (NAg) in collagen-chitosan scaffolds (CCS) for orchestrating the polarization of macrophages from the M1 inflammatory state to the M2 pro-healing state [347,348,349]. Here, NAg was applied as an antimicrobial agent and it has provided a promising therapeutic approach for burn wounds [348,349,350]. As a result, it was shown that using NAg-CCS enabled an accelerated and higher quality of wound healing with reduced expression of inflammatory factors, such as IL-6 and TNF-α, and triggered epithelialization within seven days in the wound bed. Meanwhile, the expression level of IL-10 was significantly higher in NAg-CCS compared to the control group, especially on day 4 after injury [348].

Furthermore, another natural polymer that has been widely employed in various tissue engineering applications is silk [279,281]. Silk contains two different proteins, fibroin and sericin, (Figure 5D), among which fibroin is an FDA-approved biomaterial for use in certain medical devices [281]. Silk and silk fibroin-based materials have been shaped into various hydrogels and scaffolds, such as electrospun mats, foams, sponges, microspheres, and 3D printed structures, owing to their exceptional mechanical qualities, biodegradability, and biocompatibility [281]. Several investigations have revealed that in skin burn wounds, silk materials promote reepithelization faster than traditional materials [281,351,352]. Studies also suggested that silk biomaterials induced M2 macrophages with higher anti-inflammatory cytokine profiles to promote vascularization [353,354,355]. For instance, a study assessing the effects of different mass ratios of silk fibroin (SF) and silk sericin (SS) in electrospun SF-SS fibrous films on macrophage polarization indicated that when the mass ratio of SF and SS reached 7:3, the macrophages were accompanied with high M2/M1 ratio, which displayed the highest degree of vascularization on day 14 after subcutaneous implantation in rats, and as the increased SS content, more M2c subtypes of macrophages were differentiated, showing higher IL-10 expression [353].

Natural polymers have good biological activity and have many similarities to ECM, hence, they are less likely to cause foreign body reactions [215]. Moreover, they can be degraded by enzymes after being implanted in the body, releasing immunomodulatory molecules, which can be further used to modulate macrophage polarization [277,355]. Among them, GAGs, chitosan, collagen, and silk are particularly suitable for use in tissue regeneration engineering to promote wound healing [220,279]. However, natural materials derived from animal sources have a high degree of variability with complex structures, and the extraction process is complicated and high cost [356]. Meanwhile, certain natural polymers, like polypeptides, have limited mechanical properties, whereas other natural polymers, like chitosan, have poor processability [357]. Furthermore, some scaffolds made of natural polymers have lower stability, which contributes to their higher rates of disintegration and degradation when compared to host tissue regeneration [357]. With these thematic issues in mind, developing biomaterials with appropriate mechanical, structural, degradation, and compositional qualities is important to effectively modulate macrophage polarization in tissue engineering clinical applications [356,357]. In this case, synthetic polymers have been proposed to address the issues raised above. This will be discussed in the following sections.

#### 4.1.3. Synthetic Polymers

There has been a growing interest in developing synthetic polymers for modulating macrophage polarization status in recent years [217,218,230]. Synthetic polymers can be easily developed and manufactured into batch-to-batch consistent products with particular structures [358]. Compared to natural polymers, they also have more predictable properties that allow for greater precision in tuning their physicochemical properties for various applications [217,230,358]. Synthetic polymers include degradable polymers, such as PLGA, PCL, polylactic acid (PLA), polydioxanone (PDO), and poly(urethane urea), and nondegradable polymers, such as PTFE, polyethene (PE), polyethene terephthalate (PET), polyethene glycol (PEG), polyurethane (PU), polypropylene (PP), and Poly-d-lysine (PDL) [218,230]. These polymers can be processed into hydrogels, foams, films, particles, fibers, and scaffolds, by 3D printing, electrospinning, and solid freeform fabrication, depending on their specific clinical application [358,359].

A diverse set of macrophage responses has been observed in different synthetic polymers. PTFE and PET mainly induce the production of pro-inflammatory cytokines, including TNF-α, IL-1β, IL-6, and IL-8, while PCL, PE, and PU promote more anti-inflammatory molecules such as IL-10 and CCL18, whilst PLA, PP, and poly(urethane urea) can stimulate the secretion of both pro- and anti-inflammatory modulators [218]. For instance, 3D-printed PCL scaffolds with PLLA electrospun microfibrous implanted in rats with calvarial defects increased the proportion of M2 macrophages by activating PI3K/AKT signaling pathway, enhanced angiogenesis, and accelerated new bone formation within 4 weeks implantation [360]. In another study, mice were implanted with PCL nanofibers which were coated with M2 macrophage membrane to mimic surface proteins of the natural membrane of these cells. These experiments showed marked inhibition of TLR/NF-kB/IRF-5 signaling and suppression of inflammatory genes such as IL-6, iNOS, and TNF-α compared to uncoated PCL fibers or those coated with membranes derived from M0 or M1 macrophages [361]. Zhang et al. built a 3D biodegradable waterborne PU scaffold that could act as a reservoir to store a variety of necrotic debris, cytokines, and chemokines and drive macrophages to their pores, which first polarized macrophages to the M1-like subtype to eliminate necrotic debris by upregulating hemoglobin and FoxO signaling pathways, and then induced by scar-free secreted growth factors and ECM proteins produced by inflammatory cells, the PI3K/AKT signaling pathway was activated, leading to the M2-like immune cells enriched regeneration-predominant microenvironment to promote endogenous brain regeneration following intracerebral hemorrhage [362].

Although some polymeric materials can modulate macrophage to an M1 or M2 phenotype, many materials cause classic foreign body reaction (FBR) after being inserted into the tissue [358]. Therefore, polymeric materials have been developed that are both immune-instructive and able to prevent possible complement-mediated reactions. For example, some synthetic polymer materials have been modified with natural polymers to improve their biocompatibility [230]. Wolf et al. applied ECM-coated PP mesh in the in vivo studies, which attenuated the M1 macrophage response and increased the M2/M1 (CD206/CD86) ratio following implantation within 7 days [363]. Li et al. indicated that the CS/polydopamine-modified PET graft significantly re-directed M2 macrophage polarization from M1, increased the production of pro-repair cytokines including IL-4, IL-10, and TGF-β1, and promoted the bone regeneration process, which achieved graft-bone osseointegration at month 3, with PET fibers embedded in the new bone [364].

Despite the development of specific immuno-instructive materials, the understanding of the biological-materials interactions governing the ability of a material to modulate macrophage phenotype is not well understood such that ab initio design of a material with optimal physio-chemical properties to induce a specific macrophage response is not possible. One approach to circumvent this constraint is the use of high throughput screening, where hundreds to thousands of unique polymeric formulations at a small scale can be assessed in parallel to rapidly identify materials with desirable bio-instructive properties [365]. This approach has been successfully used to identify materials that prevent bacterial biofilm formation and fungal growth and to modulate stem cell growth and differentiation [366,367,368]. Others have used this approach to investigate the immune modulatory properties of a (meth)acrylate and (meth)acrylamide polymer library [369,370]. These studies identified polymers that could promote human monocyte-derived macrophage polarization to M1-like or M2-like phenotypes in vitro which were further validated in a murine foreign body model where pro- or anti-inflammatory responses were shown by histological examination. This demonstrated that the process can identify materials on a small scale that successfully translates to larger scales by using a machine learning model, which highlighted the potential to undertake ‘‘immune-instructive’’ rational design [369].

Overall, compared to natural polymers, synthetic polymers have a few advantages, including tunable properties, endless forms, established structures, better mechanical properties, as well as predictable and reproducible physical characteristics when they are applied for modulating macrophage polarization in tissue engineering, but one drawback of synthetic biomaterials after implantation is that they lack cell adhesion sites, which affects cell attachment and proliferation on the surface of the biomaterial and may lead to FBR [356,358]. This limits the long-term functions of the implanted biomaterials and further causes their failure [358]. With the development of more precise cell characterization techniques, certain macrophage subsets have been identified and linked to more or less favorable outcomes [358]. In order to improve the biocompatibility of biomaterials and control macrophage polarization after implantation, modifying the chemical and topographical properties of biomaterials has gained wide traction as an efficient means to develop immune-instructive medical devices for a diverse set of applications [30,357]. Some of these approaches will be further discussed in the following sections.

### 4.2. Chemical and Physical Modification

Macrophage polarization is known to be influenced by a variety of factors including biomaterial chemistry, molecular weight, shape, hydrophilicity/hydrophobicity, water absorption, lubricity, surface charge and energy, degradation, and erosion processes [216]. As such, modification of these properties has been used to fine-tune the materials’ interaction with macrophages to promote better tissue integration and healing processes [216].

#### 4.2.1. Surface Topography

The surface micro- and nano-topographical features of biomaterials can directly or indirectly control macrophage functional properties by altering their adhesion, morphology, and movement on surfaces [215,363,371,372,373,374,375]. Altering the shape of cells, particularly with the use of grooves, has been demonstrated as a method to modulate macrophage response to either M1 or M2 phenotypes. For example, Luu et al. [371] were able to modulate macrophage differentiation by creating micro- and nano-patterned grooves on titanium surfaces. According to their findings, macrophage elongation was impacted by micro- and nanopatterned grooves (groove width: 0.15~50 μm) on patterned Ti substrates, where the elongation of macrophages and expression of phenotypic markers associated with a pro-healing M2 phenotype were highest on substrates with 400~500 nm wide grooves. In a gelatin methacryloyl (GelMA) hydrogel platform study, results indicated that micropatterns affected gene expression profiles, such as GIMAP6, SCFD1, and ZSWIM7, in human macrophages cultured on microgrooves/ridges and micropillars patterning on GelMA, which significantly reduced the production of TNF-α by macrophages under LPS stimulation, compared to unpatterned GelMA [374]. In the meantime, it is found that there is no significant difference in the cytokine profile expressed by macrophages between different types of patterns (microgrooves/ridges or micropillars) in this process, which shows different results from the previous study [371,374]. This demonstrated that macrophage topographical response is complex and macrophage polarization changes on specific surface patterns. Therefore, a high throughput strategy is required in further studies to investigate the impact of specific topography differences on macrophage polarization.

To explore macrophage response beyond grooves, a high throughput screening approach has been adopted, making use of algorithm-generated libraries of micro-topographies. The TopoChip platform consists of 2176 different topographies on a single chip and has been used to identify unique topographies that can modulate macrophage attachment and polarization [372]. It was found that smaller micropillars (cylindrical shapes) with a high density promoted high cell attachment and a phenotypic shift towards M2, whereas more dispersed and larger micropillars had low cell attachment and produced an M1 phenotypic shift. Specifically, micropillar diameters in the range of 5~10 μm were found to have the highest number of macrophage attachments, with the 5 μm diameter having the highest frequency of attachment and 10 μm being the upper limit before macrophage adhesion significantly decreased.

A biomimetic approach to topography has also been employed by Monteiro et al. [373] where soft lithography on PCL membranes replicated the topography of L929 cells, eggshell membranes (ESM), as well as gram-positive and gram-negative bacteria. An increase in gene expression of IL-4, Arg-1, and Siglec-1 and an underexpression of IL-6, IL-1, and CXCL-9, indicating M2 polarization, was observed after exposure to L929 and ESM PCL membranes. For the bacterial topographical membranes, the inverse was found with overexpression of inflammatory biomarkers and under-expression of IL-4 and IL-10, indicating M1 phenotype. This study demonstrates the importance of topography within macrophage cell recognition and, thus, highlights the possibility of modulating macrophage behavior through topographical modifications. An alternative biomimetic approach is the use of electrospinning to mimic the fiber configuration of ECM [376]. According to Jia et al. [375], macrophage polarization was affected by the configuration of biodegradable electrospun poly(L-lactide-ε-caprolactone) (P(LLA-CL)) nanofibers. These nanofibers were employed to construct nerve-guidance conduits, which were evaluated on macrophage polarization and nerve regeneration in a rat sciatic nerve defect model. The in vivo findings demonstrated that, in comparison to random nanofibers, aligned nanofibers stimulated more pro-healing M2 macrophages and higher Schwann cell infiltration, as well as more axon numbers, showing better peripheral nerve regeneration at 3 weeks after surgery.

#### 4.2.2. Surface Wettability and Charge

Changes in surface wettability and/or charge of biomaterials have been shown to have a major impact on macrophage phenotypes [215,216]. Hotchkiss et al. [377] cultured macrophages on seven titanium surfaces with different hydrophobic and hydrophilic conditions and roughness (roughness: 0.59~3.64 μm and surface area ratio: 39~62%). Here, the hydrophilic and hydrophobic surfaces were created by oxygen plasma cleaning and sonicating, respectively. Results showed that smooth Ti and hydrophilic rough Ti surface induced pro-inflammatory M1-like macrophage activation with increased levels of IL-1β, IL-6, and TNF-α. In contrast, hydrophilic rough Ti promoted anti-inflammatory M2-like macrophage activation, increasing levels of IL-4 and IL-10. Duan et al. [378] reported that poly-lysine-modified poly(propylene fumarate) PU films increased surface wettability and promoted M2 in an in vivo study, which is more likely through activating focal adhesion kinase (FAK) and Rho-associated protein kinase (ROCK), and downstream PI3K/Akt1/mTOR signal axis. However, Rostam et al. [219] evaluated the effects of various surface chemistries on macrophage polarization, showing that untreated hydrophobic polystyrene (PS) surface stimulated M2-like phenotype differentiation, with high expression of MR and anti-inflammatory cytokines IL-10 and CCL18, while hydrophilic O_2_ plasma-etched PS surfaces induced M1-like phenotype, as evidenced by significantly higher expression of the pro-inflammatory transcription factors STAT1 and IRF5. Together these studies suggest that wettability alone cannot be used to explain macrophage response to the chemistry of a surface.

The surface charge of biomaterials has also been reported to influence macrophage responses [265,379,380]. It is proposed that synthetic waterborne PU nanoparticles with carboxyl groups on the surface exhibited a greater extent of inhibition on M1 polarization than those with amine groups [379]. However, according to Bartneck et al. [380], poly(ethylene oxide) (PEO)-carboxyl groups modified gold nanoparticles caused an increase in pro-inflammatory IL-1β, IL-6, TNF-α, and CCL2 suggesting polarization towards M1 phenotype by affecting the TLR signaling pathway. It was also shown that PEO-hydroxyl group-decorated gold nanorods increased IL-1 and CCL2, but amine termination gold nanorods produced M2 macrophages that were anti-inflammatory. The surface charge and wettability can be combined to promote macrophages’ anti-inflammatory responses. PET surfaces coated with poly(styrene-co-benzyl N,N-diethyldithiocarbamate) and sodium salt of poly(acrylic acid) showed hydrophilic and anionic properties and were able to induce M2 macrophages with high IL-10 secretion [381]. However, the conflicting macrophage response to change observed by various studies suggests a complex underpinning biological-material interaction. Further development of the understanding of how macrophages respond to different surface chemistries is required to enable ab initio design of a chemical modification to a biomaterial to produce a specific macrophage response.

#### 4.2.3. Substrate Stiffness and Geometry

The substrate stiffness and geometry also play important roles in tissue regeneration and wound healing cascades. They can be modified to regulate the behavior of macrophages [173,215,216,218,259,263,265]. Scott et al. [382] cultured cord blood-derived macrophages on PEG-based hydrogels with different substrate stiffness (0.1, 3.4, and 10.3 kPa). Results revealed that macrophages readily changed their phenotypes following sequential administration of pro- and anti-inflammatory cytokine cocktails, demonstrating their innate plasticity. Meanwhile, anti-inflammatory phenotypes of macrophages were observed to increase with elevated substrate stiffness, which confirmed that the macrophages display predictable behaviors that can be directed and fine-tuned through combinatorial modulation of substrate physical properties and biochemical signals (e.g., cytokines). Similarly, in a different study, bone marrow-derived macrophages were cultivated on polyacrylamide hydrogels with varying substrate stiffness (approx. 2.55, 34.88, and 63.53 kPa). The findings suggested that low substrate stiffness promoted macrophages to shift to M1 macrophages, whilst higher stiffness induced M2 macrophages, through modulating ROS-initiated NF-κB pathway [383]. In another study, Camarero-Espinosa et al. developed 3D-printed dual-porosity scaffolds based on copolymers of poly(lactide-co-caprolactone) with high and low stiffness and investigated their impact on rat alveolar macrophages in vitro and following subcutaneous implantation in a rat in vivo. Their data showed that stiffer scaffolds (>40 kPa) with comparable porosities supported an M2 phenotype, while softer scaffolds (<5 kPa) resulted in an M1 phenotype, which seems to be connected to the surface spread area of cells. This was successfully transferred to an in vivo application on a rat subcutaneous model, whereby stiffer scaffolds promoted healing while softer scaffolds caused chronic inflammation after six weeks of surgery [384].

These data further support the importance of the chemical and physical properties of biomaterials on their immune-instructive properties with clear examples of how such modifications could be used to fine-tune macrophage responses and their downstream impact on tissue homeostasis [385]. However, the exact mechanisms underlying the observed responses remain largely unknown. This is an area that requires more research and will underpin the rational design of biomaterials with distinct and predictable pro or anti-inflammatory properties for various clinical applications [221].

## 5. Conclusions

Macrophages are present in all tissues and play an important role in maintaining microenvironmental homeostasis by eradicating pathogens and cellular debris, triggering adaptive immune responses, and releasing functional immune mediators during tissue repair. Timely regulation of macrophage phenotype is a crucial and potentially decisive event during tissue remodeling, as inappropriate transitions toward the M1 or M2 macrophage phenotypes may lead to deleterious consequences. Our understanding of key regulators of macrophage polarization has grown beyond cytokines to include several other biochemical and biophysical signals including the physio-chemical properties of biomaterials. Extensive research on the role of macrophages in response to various environmental stimuli is critical to progress the development of therapeutics that leverage macrophage responses and their plasticity as potent therapeutic agents. However, there remain many unanswered questions about the kinetics of macrophage polarization in complex in vivo scenarios and the cross-talk between different signals in controlling macrophage phenotype. In the context of biomaterials, it is clear that many physio-chemical properties can be potent regulators of macrophage response, and that pro- and anti-inflammatory phenotypes can be altered by changes in surface chemistry, topography, roughness and stiffness. However, the understanding of the underlying biomaterial-biological interaction is not sufficiently developed to enable the selection of particular chemical or physical properties to produce a specific macrophage response in the same way that cytokine cocktails can be selected to induce a particular biological behavior. This may be due in part to the justifiably small number of variants used in many studies or the tendency to focus on a single physio-chemical property. However, a detailed understanding of how macrophages respond to biomaterials will require consideration of multiple factors at the same time and/or the use of high throughput screening tools where a more holistic assessment of a specific material property can be assessed. Therefore, future research should focus on developing a more detailed mechanistic understanding of how different physico-chemical properties of materials control macrophage polarization. This will generate a clear set of design principles to aid the creation of a new class of immuno-instructive biomaterials and speed up the clinical translation of such novel immunomodulatory systems.

## Figures and Tables

**Table 2:** Properties of GAGs in inflammation and macrophage polarization.

GAGs		Functions in Inflammation	Macrophage Polarization	Signaling Factors	Markers
**HA**	**LMW-HA**	LMW-HA induces the expression of inflammatory cytokines and participates in inflammation, immune stimulation, cell migration, and induction of angiogenesis [300,301].	↑M1	TLR2, TLR4, NF-κB and MyD88	↑INOS, IL-2b, IL-6, TNF-α, IL-1β and CD80 [302,303,304,305]
	**HMW-HA**	HMW-HA has anti-angiogenesis, anti-inflammatory, and immunosuppressive effects [306]. In malignant cells, HMW-HA is involved maintaining the homeostasis of tumors and supporting their migration [307].	↑M2 ↓M1	JNK and p38 pathways	↑Arg-1, MRC1, TGF-β1, IL-10, IL-11, CD68 and CD163 ↓IL-6, PGE2, TNF-α, CCL2 and IL-1β [302,308,309,310]
**CS/DS**		CS mainly regulates inflammatory responses by inhibiting the release of LPS-induced pro-inflammatory factors and related enzymes [311,312]. According to the different sulphation patterns of CS, CS shows different activities in inflammation: CSA and CSE have both pro-inflammatory and anti-inflammatory activities, while CSC and CSD have anti-inflammatory activities [297,313,314,315].	↑M2 ↓M1	TLR, LPS, CD44 and NF-kB	↑TGF-β, IL-10, Arg-1 and MRC1 ↓IL-6 and TNF-α [316,317,318]
**HP/HS**		HP/HS coordinates different levels of inflammation by interacting with numerous molecules expressed on the cell surface, such as selectins and integrins, activating cytokines and chemokines, such as the interleukin family, and inhibiting the pro-inflammatory enzymes and cytotoxic mediators [319,320,321,322,323,324,325,326].	↑M1	STAT1	↑ IL-6, TNF-α, IFN, NO [327,328]
**KS**		KS regulates inflammatory responses and can be used as a new type of therapeutic compound for the treatment of inflammation damage [329,330,331,332].	↓M1	--	↓IL-12 [333]

In this table, ↑ indicates increase, and ↓ indicates reduction.

## References

[1] Martin P., Leibovich S.J. (2005). Inflammatory cells during wound repair: The good, the bad and the ugly. Trends Cell Biol..

[2] Nourshargh S., Alon R. (2014). Leukocyte migration into inflamed tissues. Immunity.

[3] Wynn T.A., Vannella K.M. (2016). Macrophages in Tissue Repair, Regeneration, and Fibrosis. Immunity.

[4] Chazaud B. (2014). Macrophages: Supportive cells for tissue repair and regeneration. Immunobiology.

[5] Oishi Y., Manabe I. (2018). Macrophages in inflammation, repair and regeneration. Int. Immunol..

[6] Hirayama D., Iida T., Nakase H. (2017). The Phagocytic Function of Macrophage-Enforcing Innate Immunity and Tissue Homeostasis. Int. J. Mol. Sci..

[7] Novak M.L., Koh T.J. (2013). Macrophage phenotypes during tissue repair. J. Leukoc. Biol..

[8] Muntjewerff E.M., Meesters L.D., van den Bogaart G. (2020). Antigen Cross-Presentation by Macrophages. Front. Immunol..

[9] Sun M., Sun L., Huang C., Chen B.-C., Zhou Z. (2019). Induction of macrophage M2b/c polarization by adipose tissue-derived mesenchymal stem cells. J. Immunol. Res..

[10] Shapouri-Moghaddam A., Mohammadian S., Vazini H., Taghadosi M., Esmaeili S.A., Mardani F., Seifi B., Mohammadi A., Afshari J.T., Sahebkar A. (2018). Macrophage plasticity, polarization, and function in health and disease. J. Cell. Physiol..

[11] Colin S., Chinetti-Gbaguidi G., Staels B. (2014). Macrophage phenotypes in atherosclerosis. Immunol. Rev..

[12] Hesketh M., Sahin K.B., West Z.E., Murray R.Z. (2017). Macrophage Phenotypes Regulate Scar Formation and Chronic Wound Healing. Int. J. Mol. Sci..

[13] Lis-López L., Bauset C., Seco-Cervera M., Cosín-Roger J. (2021). Is the Macrophage Phenotype Determinant for Fibrosis Development?. Biomedicines.

[14] Jung M., Ma Y., Iyer R.P., DeLeon-Pennell K.Y., Yabluchanskiy A., Garrett M.R., Lindsey M.L. (2017). IL-10 improves cardiac remodeling after myocardial infarction by stimulating M2 macrophage polarization and fibroblast activation. Basic. Res. Cardiol..

[15] Laskin D.L., Sunil V.R., Gardner C.R., Laskin J.D. (2011). Macrophages and tissue injury: Agents of defense or destruction?. Annu. Rev. Pharmacol. Toxicol..

[16] Liu P., Peng J., Han G.H., Ding X., Wei S., Gao G., Huang K., Chang F., Wang Y. (2019). Role of macrophages in peripheral nerve injury and repair. Neural Regen. Res..

[17] Martin K.E., Garcia A.J. (2021). Macrophage phenotypes in tissue repair and the foreign body response: Implications for biomaterial-based regenerative medicine strategies. Acta Biomater..

[18] Mantovani A., Biswas S.K., Galdiero M.R., Sica A., Locati M. (2013). Macrophage plasticity and polarization in tissue repair and remodelling. J. Pathol..

[19] Black L.M., Lever J.M., Agarwal A. (2019). Renal Inflammation and Fibrosis: A Double-edged Sword. J. Histochem. Cytochem..

[20] Yan L., Han K., Pang B., Jin H., Zhao X., Xu X., Jiang C., Cui N., Lu T., Shi J. (2021). Surfactin-reinforced gelatin methacrylate hydrogel accelerates diabetic wound healing by regulating the macrophage polarization and promoting angiogenesis. Chem. Eng. J..

[21] Brady R.V., Thamm D.H. (2023). Tumor-associated macrophages: Prognostic and therapeutic targets for cancer in humans and dogs. Front. Immunol..

[22] Müller E., Christopoulos P.F., Halder S., Lunde A., Beraki K., Speth M., Øynebråten I., Corthay A. (2017). Toll-Like Receptor Ligands and Interferon-γ Synergize for Induction of Antitumor M1 Macrophages. Front. Immunol..

[23] Yang Q., Guo N., Zhou Y., Chen J., Wei Q., Han M. (2020). The role of tumor-associated macrophages (TAMs) in tumor progression and relevant advance in targeted therapy. Acta Pharm. Sin. B.

[24] Atri C., Guerfali F.Z., Laouini D. (2018). Role of human macrophage polarization in inflammation during infectious diseases. Int. J. Mol. Sci..

[25] Muraoka D., Harada N., Hayashi T., Tahara Y., Momose F., Sawada S., Mukai S.A., Akiyoshi K., Shiku H. (2014). Nanogel-based immunologically stealth vaccine targets macrophages in the medulla of lymph node and induces potent antitumor immunity. ACS Nano.

[26] Kim Y.K., Que R., Wang S.W., Liu W.F. (2014). Modification of biomaterials with a self-protein inhibits the macrophage response. Adv. Healthc. Mater..

[27] Singh A., Talekar M., Raikar A., Amiji M. (2014). Macrophage-targeted delivery systems for nucleic acid therapy of inflammatory diseases. J. Control. Release.

[28] Kashfi K., Kannikal J., Nath N. (2021). Macrophage reprogramming and cancer therapeutics: Role of iNOS-derived NO. Cells.

[29] Navegantes K.C., de Souza Gomes R., Pereira P.A.T., Czaikoski P.G., Azevedo C.H.M., Monteiro M.C. (2017). Immune modulation of some autoimmune diseases: The critical role of macrophages and neutrophils in the innate and adaptive immunity. J. Transl. Med..

[30] Vishwakarma A., Bhise N.S., Evangelista M.B., Rouwkema J., Dokmeci M.R., Ghaemmaghami A.M., Vrana N.E., Khademhosseini A. (2016). Engineering Immunomodulatory Biomaterials to Tune the Inflammatory Response. Trends Biotechnol..

[31] Wu Y., Hirschi K.K. (2020). Tissue-Resident Macrophage Development and Function. Front. Cell Dev. Biol..

[32] Huang X., Li Y., Fu M., Xin H.-B. (2018). Polarizing macrophages in vitro. Macrophages Methods Protoc..

[33] Sreejit G., Fleetwood A.J., Murphy A.J., Nagareddy P.R. (2020). Origins and diversity of macrophages in health and disease. Clin. Transl. Immunol..

[34] Perdiguero E.G., Klapproth K., Schulz C., Busch K., de Bruijn M., Rodewald H.R., Geissmann F. (2015). The Origin of Tissue-Resident Macrophages: When an Erythro-myeloid Progenitor Is an Erythro-myeloid Progenitor. Immunity.

[35] van Furth R., Cohn Z.A. (1968). The origin and kinetics of mononuclear phagocytes. J. Exp. Med..

[36] Epelman S., Lavine K.J., Randolph G.J. (2014). Origin and functions of tissue macrophages. Immunity.

[37] Bajgar A., Krejčová G. (2023). On the origin of the functional versatility of macrophages. Front. Physiol..

[38] Lee H., Fessler M.B., Qu P., Heymann J., Kopp J.B. (2020). Macrophage polarization in innate immune responses contributing to pathogenesis of chronic kidney disease. BMC Nephrol..

[39] Yunna C., Mengru H., Lei W., Weidong C. (2020). Macrophage M1/M2 polarization. Eur. J. Pharmacol..

[40] Arango Duque G., Descoteaux A. (2014). Macrophage cytokines: Involvement in immunity and infectious diseases. Front. Immunol..

[41] Lee K.Y. (2019). M1 and M2 polarization of macrophages: A mini-review. Med. Biol. Sci. Eng..

[42] Jain S., Tran T.H., Amiji M. (2015). Macrophage repolarization with targeted alginate nanoparticles containing IL-10 plasmid DNA for the treatment of experimental arthritis. Biomaterials.

[43] Taylor P.C., Feldmann M. (2009). Anti-TNF biologic agents: Still the therapy of choice for rheumatoid arthritis. Nat. Rev. Rheumatol..

[44] Moore K.J., Sheedy F.J., Fisher E.A. (2013). Macrophages in atherosclerosis: A dynamic balance. Nat. Rev. Immunol..

[45] Saqib U., Sarkar S., Suk K., Mohammad O., Baig M.S., Savai R. (2018). Phytochemicals as modulators of M1-M2 macrophages in inflammation. Oncotarget.

[46] dos Santos Luz R.B., Nicolazzi L.H.C.N., Saraiva Camara N.O., Braga T.T., Saraiva Camara N.O., Braga T.T. (2022). Chapter 1—Macrophages: From Metchnikoff to 2020 and ahead. Macrophages in the Human Body.

[47] Hoeffel G., Ginhoux F. (2018). Fetal monocytes and the origins of tissue-resident macrophages. Cell Immunol..

[48] Cline M.J., Moore M.A. (1972). Embryonic origin of the mouse macrophage. Blood.

[49] Winkler I.G., Sims N.A., Pettit A.R., Barbier V., Nowlan B., Helwani F., Poulton I.J., van Rooijen N., Alexander K.A., Raggatt L.J. (2010). Bone marrow macrophages maintain hematopoietic stem cell (HSC) niches and their depletion mobilizes HSCs. Blood.

[50] Heideveld E., van den Akker E. (2017). Digesting the role of bone marrow macrophages on hematopoiesis. Immunobiology.

[51] Mass E. (2018). Delineating the origins, developmental programs and homeostatic functions of tissue-resident macrophages. Int. Immunol..

[52] Gentek R., Molawi K., Sieweke M.H. (2014). Tissue macrophage identity and self-renewal. Immunol. Rev..

[53] Cannon G.J., Swanson J.A. (1992). The macrophage capacity for phagocytosis. J. Cell Sci..

[54] Rosales C., Uribe-Querol E. (2017). Phagocytosis: A Fundamental Process in Immunity. BioMed Res. Int..

[55] Martinez-Pomares L., Gordon S. (2007). Antigen presentation the macrophage way. Cell.

[56] Unanue E.R. (1984). Antigen-presenting function of the macrophage. Annu. Rev. Immunol..

[57] Gordon S., Martinez F.O. (2010). Alternative activation of macrophages: Mechanism and functions. Immunity.

[58] Murray P.J. (2017). Macrophage Polarization. Annu. Rev. Physiol..

[59] Teti G., Biondo C., Beninati C. (2016). The Phagocyte, Metchnikoff, and the Foundation of Immunology. Microbiol. Spectr..

[60] Maulitz R.C. (1978). Rudolf Virchow, Julius Cohnheim and the program of pathology. Bull. Hist. Med..

[61] Silva H.M., Báfica A., Rodrigues-Luiz G.F., Chi J., Santos P.D.A., Reis B.S., Hoytema van Konijnenburg D.P., Crane A., Arifa R.D.N., Martin P. (2019). Vasculature-associated fat macrophages readily adapt to inflammatory and metabolic challenges. J. Exp. Med..

[62] Thao N.P., Cuong N.X., Luyen B.T., Quang T.H., Hanh T.T., Kim S., Koh Y.S., Nam N.H., Van Kiem P., Van Minh C. (2013). Anti-inflammatory components of the starfish Astropecten polyacanthus. Mar. Drugs.

[63] Aderem A., Underhill D.M. (1999). Mechanisms of phagocytosis in macrophages. Annu. Rev. Immunol..

[64] Uribe-Querol E., Rosales C. (2020). Phagocytosis: Our Current Understanding of a Universal Biological Process. Front. Immunol..

[65] Botelho R.J., Grinstein S. (2011). Phagocytosis. Curr. Biol..

[66] Tang D., Kang R., Coyne C.B., Zeh H.J., Lotze M.T. (2012). PAMP s and DAMP s: Signal 0s that spur autophagy and immunity. Immunol. Rev..

[67] Mueller R.B., Sheriff A., Gaipl U.S., Wesselborg S., Lauber K. (2007). Attraction of phagocytes by apoptotic cells is mediated by lysophosphatidylcholine. Autoimmunity.

[68] Segawa K., Nagata S. (2015). An apoptotic ‘eat me’signal: Phosphatidylserine exposure. Trends Cell Biol..

[69] Tsai W.-H., Shih C.-H., Feng S.-Y., Li I., Chang S.-C., Lin Y.-C., Hsu H.-C. (2014). CX3CL1 (+) microparticles mediate the chemoattraction of alveolar macrophages toward apoptotic acute promyelocytic leukemic cells. Cell. Physiol. Biochem..

[70] Sokolowski J.D., Chabanon-Hicks C.N., Han C.Z., Heffron D.S., Mandell J.W. (2014). Fractalkine is a “find-me” signal released by neurons undergoing ethanol-induced apoptosis. Front. Cell. Neurosci..

[71] Gude D.R., Alvarez S.E., Paugh S.W., Mitra P., Yu J., Griffiths R., Barbour S.E., Milstien S., Spiegel S. (2008). Apoptosis induces expression of sphingosine kinase 1 to release sphingosine-1-phosphate as a “come-and-get-me” signal. FASEB J..

[72] Elliott M.R., Chekeni F.B., Trampont P.C., Lazarowski E.R., Kadl A., Walk S.F., Park D., Woodson R.I., Ostankovich M., Sharma P. (2009). Nucleotides released by apoptotic cells act as a find-me signal to promote phagocytic clearance. Nature.

[73] Chekeni F.B., Elliott M.R., Sandilos J.K., Walk S.F., Kinchen J.M., Lazarowski E.R., Armstrong A.J., Penuela S., Laird D.W., Salvesen G.S. (2010). Pannexin 1 channels mediate ‘find-me’signal release and membrane permeability during apoptosis. Nature.

[74] Ravichandran K.S. (2010). Find-me and eat-me signals in apoptotic cell clearance: Progress and conundrums. J. Exp. Med..

[75] Mosser D.M., Zhang X. (2011). Measuring opsonic phagocytosis via Fcγ receptors and complement receptors on macrophages. Curr. Protoc. Immunol..

[76] Barth N.D., Marwick J.A., Vendrell M., Rossi A.G., Dransfield I. (2017). The “phagocytic synapse” and clearance of apoptotic cells. Front. Immunol..

[77] Kuhlman M., Joiner K., Ezekowitz R. (1989). The human mannose-binding protein functions as an opsonin. J. Exp. Med..

[78] Areschoug T., Gordon S. (2009). Scavenger receptors: Role in innate immunity and microbial pathogenesis. Cell. Microbiol..

[79] Palecanda A., Kobzik L. (2001). Receptors for unopsonized particles: The role of alveolar macrophage scavenger receptors. Curr. Mol. Med..

[80] Silverstein R.L., Febbraio M. (2009). CD36, a scavenger receptor involved in immunity, metabolism, angiogenesis, and behavior. Sci. Signal..

[81] Canton J., Neculai D., Grinstein S. (2013). Scavenger receptors in homeostasis and immunity. Nat. Rev. Immunol..

[82] Richardson M.B., Williams S.J. (2014). MCL and Mincle: C-type lectin receptors that sense damaged self and pathogen-associated molecular patterns. Front. Immunol..

[83] Mayer S., Raulf M.-K., Lepenies B. (2017). C-type lectins: Their network and roles in pathogen recognition and immunity. Histochem. Cell Biol..

[84] Albacker L.A., Yu S., Bedoret D., Lee W.L., Umetsu S.E., Monahan S., Freeman G.J., Umetsu D.T., DeKruyff R.H. (2013). TIM-4, expressed by medullary macrophages, regulates respiratory tolerance by mediating phagocytosis of antigen-specific T cells. Mucosal Immunol..

[85] Park D., Tosello-Trampont A.-C., Elliott M.R., Lu M., Haney L.B., Ma Z., Klibanov A.L., Mandell J.W., Ravichandran K.S. (2007). BAI1 is an engulfment receptor for apoptotic cells upstream of the ELMO/Dock180/Rac module. Nature.

[86] Lemke G., Burstyn-Cohen T. (2010). TAM receptors and the clearance of apoptotic cells. Ann. N. Y Acad. Sci..

[87] Burstyn-Cohen T., Fresia R. (2023). TAM receptors in phagocytosis: Beyond the mere internalization of particles. Immunol. Rev..

[88] Poirier M.B., Fiorino C., Rajasekar T.K., Harrison R.E. (2020). F-actin flashes on phagosomes mechanically deform contents for efficient digestion in macrophages. J. Cell Sci..

[89] Dingjan I., Linders P.T.A., Verboogen D.R.J., Revelo N.H., Ter Beest M., van den Bogaart G. (2018). Endosomal and Phagosomal SNAREs. Physiol. Rev..

[90] Hatsuzawa K., Tamura T., Hashimoto H., Hashimoto H., Yokoya S., Miura M., Nagaya H., Wada I. (2006). Involvement of Syntaxin 18, an Endoplasmic Reticulum (ER)-localized SNARE Protein, in ER-mediated Phagocytosis. Mol. Biol. Cell.

[91] Tjelle T.E., Lovdal T., Berg T. (2000). Phagosome dynamics and function. Bioessays.

[92] Canton J. (2014). Phagosome maturation in polarized macrophages. J. Leukoc. Biol..

[93] Fountain A., Inpanathan S., Alves P., Verdawala M.B., Botelho R.J. (2021). Phagosome maturation in macrophages: Eat, digest, adapt, and repeat. Adv. Biol. Regul..

[94] Stuart L.M., Ezekowitz R.A. (2005). Phagocytosis: Elegant complexity. Immunity.

[95] Kloc M., Uosef A., Kubiak J.Z., Ghobrial R.M. (2020). Macrophage Proinflammatory Responses to Microorganisms and Transplanted Organs. Int. J. Mol. Sci..

[96] Banga S., Gao P., Shen X., Fiscus V., Zong W.-X., Chen L., Luo Z.-Q. (2007). *Legionella pneumophila* inhibits macrophage apoptosis by targeting pro-death members of the Bcl2 protein family. Proc. Natl. Acad. Sci. USA.

[97] Lanzavecchia A. (1985). Antigen-specific interaction between T and B cells. Nature.

[98] den Haan J.M., Arens R., van Zelm M.C. (2014). The activation of the adaptive immune system: Cross-talk between antigen-presenting cells, T cells and B cells. Immunol. Lett..

[99] Phan T.G., Green J.A., Gray E.E., Xu Y., Cyster J.G. (2009). Immune complex relay by subcapsular sinus macrophages and noncognate B cells drives antibody affinity maturation. Nat. Immunol..

[100] Gonzalez S.F., Degn S.E., Pitcher L.A., Woodruff M., Heesters B.A., Carroll M.C. (2011). Trafficking of B cell antigen in lymph nodes. Annu. Rev. Immunol..

[101] Junt T., Moseman E.A., Iannacone M., Massberg S., Lang P.A., Boes M., Fink K., Henrickson S.E., Shayakhmetov D.M., Di Paolo N.C. (2007). Subcapsular sinus macrophages in lymph nodes clear lymph-borne viruses and present them to antiviral B cells. Nature.

[102] Carrasco Y.R., Batista F.D. (2007). B cells acquire particulate antigen in a macrophage-rich area at the boundary between the follicle and the subcapsular sinus of the lymph node. Immunity.

[103] Moran I., Grootveld A.K., Nguyen A., Phan T.G. (2019). Subcapsular Sinus Macrophages: The Seat of Innate and Adaptive Memory in Murine Lymph Nodes. Trends Immunol..

[104] Martinez-Pomares L., Gordon S. (2012). CD169+ macrophages at the crossroads of antigen presentation. Trends Immunol..

[105] Kuka M., Iannacone M. (2014). The role of lymph node sinus macrophages in host defense. Ann. N. Y. Acad. Sci..

[106] Louie D.A.P., Liao S. (2019). Lymph node subcapsular sinus macrophages as the frontline of lymphatic immune defense. Front. Immunol..

[107] Unanue E.R., Cerottini J.C., Bedford M. (1969). Persistence of Antigen on the Surface of Macrophages. Nature.

[108] Taylor P.R., Martinez-Pomares L., Stacey M., Lin H.H., Brown G.D., Gordon S. (2005). Macrophage receptors and immune recognition. Annu. Rev. Immunol..

[109] Phan T.G., Grigorova I., Okada T., Cyster J.G. (2007). Subcapsular encounter and complement-dependent transport of immune complexes by lymph node B cells. Nat. Immunol..

[110] Mantegazza A.R., Magalhaes J.G., Amigorena S., Marks M.S. (2013). Presentation of phagocytosed antigens by MHC class I and II. Traffic.

[111] Neefjes J., Jongsma M.L.M., Paul P., Bakke O. (2011). Towards a systems understanding of MHC class I and MHC class II antigen presentation. Nat. Rev. Immunol..

[112] Kotsias F., Cebrian I., Alloatti A. (2019). Antigen processing and presentation. Int. Rev. Cell Mol. Biol..

[113] Bevan M.J. (1995). Antigen presentation to cytotoxic T lymphocytes in vivo. J. Exp. Med..

[114] Brutkiewicz R.R., Lin Y., Cho S., Hwang Y.K., Sriram V., Roberts T.J. (2003). CD1d-mediated antigen presentation to natural killer T (NKT) cells. Crit. Rev. Immunol..

[115] Cruz-Leal Y., Grubaugh D., Nogueira C.V., Lopetegui-González I., Del Valle A., Escalona F., Laborde R.J., Alvarez C., Fernández L.E., Starnbach M.N. (2018). The vacuolar pathway in macrophages plays a major role in antigen cross-presentation induced by the pore-forming protein sticholysin II encapsulated into liposomes. Front. Immunol..

[116] Grabowska J., Lopez-Venegas M.A., Affandi A.J., den Haan J.M.M. (2018). CD169(+) Macrophages Capture and Dendritic Cells Instruct: The Interplay of the Gatekeeper and the General of the Immune System. Front. Immunol..

[117] Schliehe C., Redaelli C., Engelhardt S., Fehlings M., Mueller M., van Rooijen N., Thiry M., Hildner K., Weller H., Groettrup M. (2011). CD8- dendritic cells and macrophages cross-present poly(D,L-lactate-co-glycolate) acid microsphere-encapsulated antigen in vivo. J. Immunol..

[118] Hey Y.Y., Tan J.K., O’Neill H.C. (2015). Redefining Myeloid Cell Subsets in Murine Spleen. Front. Immunol..

[119] Enders M., Franken L., Philipp M.S., Kessler N., Baumgart A.K., Eichler M., Wiertz E.J.H., Garbi N., Kurts C. (2020). Splenic Red Pulp Macrophages Cross-Prime Early Effector CTL That Provide Rapid Defense against Viral Infections. J. Immunol..

[120] Tang-Huau T.-L., Gueguen P., Goudot C., Durand M., Bohec M., Baulande S., Pasquier B., Amigorena S., Segura E. (2018). Human in vivo-generated monocyte-derived dendritic cells and macrophages cross-present antigens through a vacuolar pathway. Nat. Commun..

[121] Ruedl C., Storni T., Lechner F., Bächi T., Bachmann M.F. (2002). Cross-presentation of virus-like particles by skin-derived CD8–dendritic cells: A dispensable role for TAP. Eur. J. Immunol..

[122] Backer R., Schwandt T., Greuter M., Oosting M., Jüngerkes F., Tüting T., Boon L., O’Toole T., Kraal G., Limmer A. (2010). Effective collaboration between marginal metallophilic macrophages and CD8+ dendritic cells in the generation of cytotoxic T cells. Proc. Natl. Acad. Sci. USA.

[123] van Dinther D., Veninga H., Iborra S., Borg E.G.F., Hoogterp L., Olesek K., Beijer M.R., Schetters S.T.T., Kalay H., Garcia-Vallejo J.J. (2018). Functional CD169 on Macrophages Mediates Interaction with Dendritic Cells for CD8(+) T Cell Cross-Priming. Cell Rep..

[124] Blohm U., Roth E., Brommer K., Dumrese T., Rosenthal F.M., Pircher H. (2002). Lack of effector cell function and altered tetramer binding of tumor-infiltrating lymphocytes. J. Immunol..

[125] Hoeve M.A., Savage N.D.L., de Boer T., Langenberg D.M.L., de Waal Malefyt R., Ottenhoff T.H.M., Verreck F.A.W. (2006). Divergent effects of IL-12 and IL-23 on the production of IL-17 by human T cells. Eur. J. Immunol..

[126] Riabov V., Gudima A., Wang N., Mickley A., Orekhov A., Kzhyshkowska J. (2014). Role of tumor associated macrophages in tumor angiogenesis and lymphangiogenesis. Front. Physiol..

[127] Modak M., Mattes A.K., Reiss D., Skronska-Wasek W., Langlois R., Sabarth N., Konopitzky R., Ramirez F., Lehr K., Mayr T. (2022). CD206+ tumor-associated macrophages cross-present tumor antigen and drive antitumor immunity. JCI Insight.

[128] Di Gioacchino M., Della Valle L., Allegra A., Pioggia G., Gangemi S. (2022). AllergoOncology: Role of immune cells and immune proteins. Clin. Transl. Allergy.

[129] Germano G., Frapolli R., Belgiovine C., Anselmo A., Pesce S., Liguori M., Erba E., Uboldi S., Zucchetti M., Pasqualini F. (2013). Role of macrophage targeting in the antitumor activity of trabectedin. Cancer Cell.

[130] Mosser D.M., Edwards J.P. (2008). Exploring the full spectrum of macrophage activation. Nat. Rev. Immunol..

[131] Li M., Hou Q., Zhong L., Zhao Y., Fu X. (2021). Macrophage Related Chronic Inflammation in Non-Healing Wounds. Front. Immunol..

[132] Lu Y.-C., Yeh W.-C., Ohashi P.S. (2008). LPS/TLR4 signal transduction pathway. Cytokine.

[133] Akira S., Takeda K. (2004). Toll-like receptor signalling. Nat. Rev. Immunol..

[134] Koo S.-J., Garg N.J. (2019). Metabolic programming of macrophage functions and pathogens control. Redox Biol..

[135] Jin J., Xiao Y., Hu H., Zou Q., Li Y., Gao Y., Ge W., Cheng X., Sun S.-C. (2015). Proinflammatory TLR signalling is regulated by a TRAF2-dependent proteolysis mechanism in macrophages. Nat. Commun..

[136] Takeuch O., Akira S. (2011). Epigenetic control of macrophage polarization. Eur. J. Immunol..

[137] Wajant H., Scheurich P. (2011). TNFR1-induced activation of the classical NF-κB pathway. FEBS J..

[138] Kroner A., Greenhalgh A.D., Zarruk J.G., Dos Santos R.P., Gaestel M., David S. (2014). TNF and increased intracellular iron alter macrophage polarization to a detrimental M1 phenotype in the injured spinal cord. Neuron.

[139] Bi Y., Zhou J., Yang H., Wang X., Zhang X., Wang Q., Wu X., Han Y., Song Y., Tan Y. (2014). IL-17A Produced by Neutrophils Protects against Pneumonic Plague through Orchestrating IFN-γ–Activated Macrophage Programming. J. Immunol..

[140] Watkins S.K., Egilmez N.K., Suttles J., Stout R.D. (2007). IL-12 rapidly alters the functional profile of tumor-associated and tumor-infiltrating macrophages in vitro and in vivo. J. Immunol..

[141] Fleetwood A.J., Dinh H., Cook A.D., Hertzog P.J., Hamilton J.A. (2009). GM-CSF-and M-CSF-dependent macrophage phenotypes display differential dependence on type I interferon signaling. J. Leukoc. Biol..

[142] Seok S.H., Heo J.-I., Hwang J.-H., Na Y.-R., Yun J.-H., Lee E.H., Park J.-W., Cho C.-H. (2013). Angiopoietin-1 elicits pro-inflammatory responses in monocytes and differentiating macrophages. Mol. Cells.

[143] Kerneur C., Cano C.E., Olive D. (2022). Major pathways involved in macrophage polarization in cancer. Front. Immunol..

[144] Watford W.T., Moriguchi M., Morinobu A., O’Shea J.J. (2003). The biology of IL-12: Coordinating innate and adaptive immune responses. Cytokine Growth Factor Rev..

[145] Wojno E.D.T., Hunter C.A., Stumhofer J.S. (2019). The immunobiology of the interleukin-12 family: Room for discovery. Immunity.

[146] Xu J., Chi F., Tsukamoto H. (2015). Notch signaling and M1 macrophage activation in obesity-alcohol synergism. Clin. Res. Hepatol. Gastroenterol..

[147] Zhu L., Zhao Q., Yang T., Ding W., Zhao Y. (2015). Cellular metabolism and macrophage functional polarization. Int. Rev. Immunol..

[148] Hall C.J., Boyle R.H., Astin J.W., Flores M.V., Oehlers S.H., Sanderson L.E., Ellett F., Lieschke G.J., Crosier K.E., Crosier P.S. (2013). Immunoresponsive gene 1 augments bactericidal activity of macrophage-lineage cells by regulating β-oxidation-dependent mitochondrial ROS production. Cell Metab..

[149] Wilson H.M. (2014). SOCS Proteins in Macrophage Polarization and Function. Front. Immunol..

[150] Lawrence T., Natoli G. (2011). Transcriptional regulation of macrophage polarization: Enabling diversity with identity. Nat. Rev. Immunol..

[151] Chen X., Dou J., Fu Z., Qiu Y., Zou L., Huang D., Tan X. (2022). Macrophage M1 polarization mediated via the IL-6/STAT3 pathway contributes to apical periodontitis induced by Porphyromonas gingivalis. J. Appl. Oral. Sci..

[152] Yang T., Wang R., Liu H., Wang L., Li J., Wu S., Chen X., Yang X., Zhao Y. (2021). Berberine regulates macrophage polarization through IL-4-STAT6 signaling pathway in Helicobacter pylori-induced chronic atrophic gastritis. Life Sci..

[153] Zhang W., Xu W., Xiong S. (2011). Macrophage differentiation and polarization via phosphatidylinositol 3-kinase/Akt-ERK signaling pathway conferred by serum amyloid P component. J. Immunol..

[154] Wang L.-X., Zhang S.-X., Wu H.-J., Rong X.-L., Guo J. (2019). M2b macrophage polarization and its roles in diseases. J. Leukoc. Biol..

[155] Mohapatra S., Pioppini C., Ozpolat B., Calin G.A. (2021). Non-coding RNAs regulation of macrophage polarization in cancer. Mol. Cancer.

[156] Sureshbabu A., Muhsin S.A., Choi M.E. (2016). TGF-β signaling in the kidney: Profibrotic and protective effects. Am. J. Physiol. -Ren. Physiol..

[157] Mancino A., Lawrence T. (2010). Nuclear factor-κB and tumor-associated macrophages. Clin. Cancer Res..

[158] Chen L., Wang S., Wang Y., Zhang W., Ma K., Hu C., Zhu H., Liang S., Liu M., Xu N. (2018). IL-6 influences the polarization of macrophages and the formation and growth of colorectal tumor. Oncotarget.

[159] Bai Y., Zhang X., Zhou J., Guo J., Liu Y., Liang C., Wang W., Xing Y., Wu J., Hu D. (2023). A2aR on lung adenocarcinoma cells: A novel target for cancer therapy via recruiting and regulating tumor-associated macrophages. Chem.-Biol. Interact..

[160] Fadok V.A., Bratton D.L., Konowal A., Freed P.W., Westcott J.Y., Henson P.M. (1998). Macrophages that have ingested apoptotic cells in vitro inhibit proinflammatory cytokine production through autocrine/paracrine mechanisms involving TGF-beta, PGE2, and PAF. J. Clin. Investig..

[161] Graney P., Ben-Shaul S., Landau S., Bajpai A., Singh B., Eager J., Cohen A., Levenberg S., Spiller K. (2020). Macrophages of diverse phenotypes drive vascularization of engineered tissues. Sci. Adv..

[162] Yang Z., Min Z., Yu B. (2020). Reactive oxygen species and immune regulation. Int. Rev. Immunol..

[163] Mills C.D. (2015). Anatomy of a discovery: m1 and m2 macrophages. Front. Immunol..

[164] Mills C. (2012). M1 and M2 macrophages: Oracles of health and disease. Crit. Rev. Immunol..

[165] Zhang M., He Y., Sun X., Li Q., Wang W., Zhao A., Di W. (2014). A high M1/M2 ratio of tumor-associated macrophages is associated with extended survival in ovarian cancer patients. J. Ovarian Res..

[166] Macciò A., Gramignano G., Cherchi M.C., Tanca L., Melis L., Madeddu C. (2020). Role of M1-polarized tumor-associated macrophages in the prognosis of advanced ovarian cancer patients. Sci. Rep..

[167] Li Y., Cao F., Li M., Li P., Yu Y., Xiang L., Xu T., Lei J., Tai Y.Y., Zhu J. (2018). Hydroxychloroquine induced lung cancer suppression by enhancing chemo-sensitization and promoting the transition of M2-TAMs to M1-like macrophages. J. Exp. Clin. Cancer Res..

[168] Yuan C., Yang D., Ma J., Yang J., Xue J., Song F., Liu X. (2020). Modulation of Wnt/β-catenin signaling in IL-17A-mediated macrophage polarization of RAW264.7 cells. Braz. J. Med. Biol. Res..

[169] Wang Q., Cheng F., Ma T.-t., Xiong H.-Y., Li Z.-W., Xie C.-L., Liu C.-Y., Tu Z.-G. (2016). Interleukin-12 inhibits the hepatocellular carcinoma growth by inducing macrophage polarization to the M1-like phenotype through downregulation of Stat-3. Mol. Cell. Biochem..

[170] Hamilton J.A. (2008). Colony-stimulating factors in inflammation and autoimmunity. Nat. Rev. Immunol..

[171] Jablonski K.A., Amici S.A., Webb L.M., Ruiz-Rosado Jde D., Popovich P.G., Partida-Sanchez S., Guerau-de-Arellano M. (2015). Novel Markers to Delineate Murine M1 and M2 Macrophages. PLoS ONE.

[172] Martinez F.O., Gordon S. (2014). The M1 and M2 paradigm of macrophage activation: Time for reassessment. F1000Prime Rep..

[173] Alvarez M.M., Liu J.C., Trujillo-de Santiago G., Cha B.H., Vishwakarma A., Ghaemmaghami A.M., Khademhosseini A. (2016). Delivery strategies to control inflammatory response: Modulating M1-M2 polarization in tissue engineering applications. J. Control. Release.

[174] Lu J., Cao Q., Zheng D., Sun Y., Wang C., Yu X., Wang Y., Lee V.W., Zheng G., Tan T.K. (2013). Discrete functions of M2a and M2c macrophage subsets determine their relative efficacy in treating chronic kidney disease. Kidney Int..

[175] Oates T.C., Moura P.L., Cross S., Roberts K., Baum H.E., Haydn-Smith K.L., Wilson M.C., Heesom K.J., Severn C.E., Toye A.M. (2023). Defining the proteomic landscape of cultured macrophages and their polarization continuum. Immunol. Cell Biol..

[176] Yao Y., Xu X.-H., Jin L. (2019). Macrophage polarization in physiological and pathological pregnancy. Front. Immunol..

[177] Amici S.A., Young N.A., Narvaez-Miranda J., Jablonski K.A., Arcos J., Rosas L., Papenfuss T.L., Torrelles J.B., Jarjour W.N., Guerau-de-Arellano M. (2018). CD38 is robustly induced in human macrophages and monocytes in inflammatory conditions. Front. Immunol..

[178] Meng X.-M., Tang P.M.-K., Li J., Lan H.Y. (2015). Macrophage phenotype in kidney injury and repair. Kidney Dis..

[179] Dreschers S., Ohl K., Lehrke M., Möllmann J., Denecke B., Costa I., Vogl T., Viemann D., Roth J., Orlikowsky T. (2019). Impaired cellular energy metabolism in cord blood macrophages contributes to abortive response toward inflammatory threats. Nat. Commun..

[180] Lateef Z., Stuart G., Jones N., Mercer A., Fleming S., Wise L. (2019). The cutaneous inflammatory response to thermal burn injury in a murine model. Int. J. Mol. Sci..

[181] Apostolopoulos V., De Courten M.P., Stojanovska L., Blatch G.L., Tangalakis K., De Courten B. (2016). The complex immunological and inflammatory network of adipose tissue in obesity. Mol. Nutr. Food Res..

[182] Sasaki A. (2017). Microglia and brain macrophages: An update. Neuropathology.

[183] Kong Q., Li N., Cheng H., Zhang X., Cao X., Qi T., Dai L., Zhang Z., Chen X., Li C. (2019). HSPA12A is a novel player in nonalcoholic steatohepatitis via promoting nuclear PKM2-mediated M1 macrophage polarization. Diabetes.

[184] Elchaninov A., Lokhonina A., Vishnyakova P., Soboleva A., Poltavets A., Artemova D., Makarov A., Glinkina V., Goldshtein D., Bolshakova G. (2021). Marco+ macrophage dynamics in regenerating liver after 70% liver resection in mice. Biomedicines.

[185] Tawakol A., Singh P., Mojena M., Pimentel-Santillana M., Emami H., MacNabb M., Rudd J.H., Narula J., Enriquez J.A., Través P.G. (2015). HIF-1α and PFKFB3 mediate a tight relationship between proinflammatory activation and anerobic metabolism in atherosclerotic macrophages. Arterioscler. Thromb. Vasc. Biol..

[186] Wu R., Chen F., Wang N., Tang D., Kang R. (2020). ACOD1 in immunometabolism and disease. Cell. Mol. Immunol..

[187] Zhang M.-Z., Wang X., Wang Y., Niu A., Wang S., Zou C., Harris R.C. (2017). IL-4/IL-13–mediated polarization of renal macrophages/dendritic cells to an M2a phenotype is essential for recovery from acute kidney injury. Kidney Int..

[188] Raimondo T.M., Mooney D.J. (2018). Functional muscle recovery with nanoparticle-directed M2 macrophage polarization in mice. Proc. Natl. Acad. Sci. USA.

[189] Nelms K., Keegan A.D., Zamorano J., Ryan J.J., Paul W.E. (1999). The IL-4 receptor: Signaling mechanisms and biologic functions. Annu. Rev. Immunol..

[190] Nelson M.P., Christmann B.S., Werner J.L., Metz A.E., Trevor J.L., Lowell C.A., Steele C. (2011). IL-33 and M2a alveolar macrophages promote lung defense against the atypical fungal pathogen Pneumocystis murina. J. Immunol..

[191] Mazher M., Moqidem Y.A., Zidan M., Sayed A.A., Abdellatif A. (2023). Autophagic reprogramming of bone marrow–derived macrophages. Immunol. Res..

[192] Little A.C., Pathanjeli P., Wu Z., Bao L., Goo L.E., Yates J.A., Oliver C.R., Soellner M.B., Merajver S.D. (2019). IL-4/IL-13 stimulated macrophages enhance breast cancer invasion via rho-GTPase regulation of synergistic VEGF/CCL-18 signaling. Front. Oncol..

[193] Rőszer T. (2015). Understanding the mysterious M2 macrophage through activation markers and effector mechanisms. Mediat. Inflamm..

[194] Tseng W.-C., Tsai M.-T., Chen N.-J., Tarng D.-C. (2020). Trichostatin A alleviates renal interstitial fibrosis through modulation of the M2 macrophage subpopulation. Int. J. Mol. Sci..

[195] Hsieh S.W., Huang L.C., Chang Y.P., Hung C.H., Yang Y.H. (2020). M2b macrophage subset decrement as an indicator of cognitive function in Alzheimer’s disease. Psychiatry Clin. Neurosci..

[196] Lee C.-H., Choi E. (2018). Macrophages and Inflammation. J. Rheum. Dis..

[197] Suzuki K., Meguro K., Nakagomi D., Nakajima H. (2017). Roles of alternatively activated M2 macrophages in allergic contact dermatitis. Allergol. Int..

[198] Yang R., Liao Y., Wang L., He P., Hu Y., Yuan D., Wu Z., Sun X. (2019). Exosomes derived from M2b macrophages attenuate DSS-induced colitis. Front. Immunol..

[199] Koscsó B., Csóka B., Kókai E., Németh Z.H., Pacher P., Virág L., Leibovich S.J., Haskó G. (2013). Adenosine augments IL-10-induced STAT3 signaling in M2c macrophages. J. Leukoc. Biol..

[200] Lurier E.B., Dalton D., Dampier W., Raman P., Nassiri S., Ferraro N.M., Rajagopalan R., Sarmady M., Spiller K.L. (2017). Transcriptome analysis of IL-10-stimulated (M2c) macrophages by next-generation sequencing. Immunobiology.

[201] Hao N.-B., Lü M.-H., Fan Y.-H., Cao Y.-L., Zhang Z.-R., Yang S.-M. (2012). Macrophages in Tumor Microenvironments and the Progression of Tumors. Clin. Dev. Immunol..

[202] Stempin C.C., Dulgerian L.R., Garrido V.V., Cerban F.M. (2010). Arginase in parasitic infections: Macrophage activation, immunosuppression, and intracellular signals. J. Biomed. Biotechnol..

[203] Ferrante C.J., Pinhal-Enfield G., Elson G., Cronstein B.N., Hasko G., Outram S., Leibovich S.J. (2013). The adenosine-dependent angiogenic switch of macrophages to an M2-like phenotype is independent of interleukin-4 receptor alpha (IL-4Rα) signaling. Inflammation.

[204] Wang J., Li D., Cang H., Guo B. (2019). Crosstalk between cancer and immune cells: Role of tumor-associated macrophages in the tumor microenvironment. Cancer Med..

[205] Xiao W., Yang Y., Chu C., Rung S.-A., Wang Z., Man Y., Lin J., Qu Y. (2023). Macrophage response mediated by extracellular matrix: Recent progress. Biomed. Mater..

[206] Hong H., Tian X.Y. (2020). The role of macrophages in vascular repair and regeneration after ischemic injury. Int. J. Mol. Sci..

[207] Chawla A. (2010). Control of macrophage activation and function by PPARs. Circ. Res..

[208] Colegio O.R., Chu N.Q., Szabo A.L., Chu T., Rhebergen A.M., Jairam V., Cyrus N., Brokowski C.E., Eisenbarth S.C., Phillips G.M. (2014). Functional polarization of tumour-associated macrophages by tumour-derived lactic acid. Nature.

[209] Mantovani A., Sica A., Locati M. (2005). Macrophage polarization comes of age. Immunity.

[210] Tu G.-w., Shi Y., Zheng Y.-j., Ju M.-j., He H.-y., Ma G.-g., Hao G.-w., Luo Z. (2017). Glucocorticoid attenuates acute lung injury through induction of type 2 macrophage. J. Transl. Med..

[211] Zhang W., Chen L., Ma K., Zhao Y., Liu X., Wang Y., Liu M., Liang S., Zhu H., Xu N. (2016). Polarization of macrophages in the tumor microenvironment is influenced by EGFR signaling within colon cancer cells. Oncotarget.

[212] Liu F., Qiu H., Xue M., Zhang S., Zhang X., Xu J., Chen J., Yang Y., Xie J. (2019). MSC-secreted TGF-β regulates lipopolysaccharide-stimulated macrophage M2-like polarization via the Akt/FoxO1 pathway. Stem Cell Res. Ther..

[213] Braune J., Weyer U., Hobusch C., Mauer J., Brüning J.C., Bechmann I., Gericke M. (2017). IL-6 regulates M2 polarization and local proliferation of adipose tissue macrophages in obesity. J. Immunol..

[214] Coffelt S.B., Tal A.O., Scholz A., De Palma M., Patel S., Urbich C., Biswas S.K., Murdoch C., Plate K.H., Reiss Y. (2010). Angiopoietin-2 regulates gene expression in TIE2-expressing monocytes and augments their inherent proangiogenic functions. Cancer Res..

[215] Mao J., Chen L., Cai Z., Qian S., Liu Z., Zhao B., Zhang Y., Sun X., Cui W. (2022). Advanced Biomaterials for Regulating Polarization of Macrophages in Wound Healing. Adv. Funct. Mater..

[216] Sridharan R., Cameron A.R., Kelly D.J., Kearney C.J., O’Brien F.J. (2015). Biomaterial based modulation of macrophage polarization: A review and suggested design principles. Mater. Today.

[217] Liu Y., Segura T. (2020). Biomaterials-Mediated Regulation of Macrophage Cell Fate. Front. Bioeng. Biotechnol..

[218] Boersema G.S., Grotenhuis N., Bayon Y., Lange J.F., Bastiaansen-Jenniskens Y.M. (2016). The Effect of Biomaterials Used for Tissue Regeneration Purposes on Polarization of Macrophages. Biores Open Access.

[219] Rostam H.M., Singh S., Salazar F., Magennis P., Hook A., Singh T., Vrana N.E., Alexander M.R., Ghaemmaghami A.M. (2016). The impact of surface chemistry modification on macrophage polarisation. Immunobiology.

[220] Li Z., Bratlie K.M. (2021). The Influence of Polysaccharides-Based Material on Macrophage Phenotypes. Macromol. Biosci..

[221] Brown B.N., Ratner B.D., Goodman S.B., Amar S., Badylak S.F. (2012). Macrophage polarization: An opportunity for improved outcomes in biomaterials and regenerative medicine. Biomaterials.

[222] Saclier M., Yacoub-Youssef H., Mackey A.L., Arnold L., Ardjoune H., Magnan M., Sailhan F., Chelly J., Pavlath G.K., Mounier R. (2013). Differentially activated macrophages orchestrate myogenic precursor cell fate during human skeletal muscle regeneration. Stem Cells.

[223] Zhang F., Wang H., Wang X., Jiang G., Liu H., Zhang G., Wang H., Fang R., Bu X., Cai S. (2016). TGF-β induces M2-like macrophage polarization via SNAIL-mediated suppression of a pro-inflammatory phenotype. Oncotarget.

[224] Ji X., Lei Z., Yuan M., Zhu H., Yuan X., Liu W., Pu H., Jiang J., Zhang Y., Jiang X. (2020). Cartilage repair mediated by thermosensitive photocrosslinkable TGFβ1-loaded GM-HPCH via immunomodulating macrophages, recruiting MSCs and promoting chondrogenesis. Theranostics.

[225] Wang Q., Li H., Xiao Y., Li S., Li B., Zhao X., Ye L., Guo B., Chen X., Ding Y. (2015). Locally controlled delivery of TNFα antibody from a novel glucose-sensitive scaffold enhances alveolar bone healing in diabetic conditions. J. Control. Release.

[226] Liu L., Zhang J., Li Z., Yang Y., Li L., Zhao Y., Zhao J. (2021). Enzyme-Loaded Catalytic Macrophage Vesicles with Cascade Amplification of Tumor-Targeting for Oxygenated Photodynamic Therapy. Int. J. Nanomed..

[227] Guiducci C., Vicari A.P., Sangaletti S., Trinchieri G., Colombo M.P. (2005). Redirecting in vivo elicited tumor infiltrating macrophages and dendritic cells towards tumor rejection. Cancer Res..

[228] He W., Kapate N., Shields C.W., Mitragotri S. (2020). Drug delivery to macrophages: A review of targeting drugs and drug carriers to macrophages for inflammatory diseases. Adv. Drug Deliv. Rev..

[229] Wen D., Chen G., Chen Q., Li P.Y., Cheng H., Gu Z. (2019). Engineering protein delivery depots for cancer immunotherapy. Bioconjugate Chem..

[230] Nair L.S., Laurencin C.T. (2007). Biodegradable polymers as biomaterials. Progress. Polym. Sci..

[231] Ezike T.C., Okpala U.S., Onoja U.L., Nwike C.P., Ezeako E.C., Okpara O.J., Okoroafor C.C., Eze S.C., Kalu O.L., Odoh E.C. (2023). Advances in drug delivery systems, challenges and future directions. Heliyon.

[232] Tabata K., Kurosaka S., Watanabe M., Edamura K., Satoh T., Yang G., Abdelfattah E., Wang J., Goltsov A., Floryk D. (2011). Tumor growth and metastasis suppression by Glipr1 gene-modified macrophages in a metastatic prostate cancer model. Gene Ther..

[233] Boehler R.M., Kuo R., Shin S., Goodman A.G., Pilecki M.A., Gower R.M., Leonard J.N., Shea L.D. (2014). Lentivirus delivery of IL-10 to promote and sustain macrophage polarization towards an anti-inflammatory phenotype. Biotechnol. Bioeng..

[234] Nikitina E., Larionova I., Choinzonov E., Kzhyshkowska J. (2018). Monocytes and Macrophages as Viral Targets and Reservoirs. Int. J. Mol. Sci..

[235] Reid T., Warren R., Kirn D. (2002). Intravascular adenoviral agents in cancer patients: Lessons from clinical trials. Cancer Gene Ther..

[236] Raper S.E., Chirmule N., Lee F.S., Wivel N.A., Bagg A., Gao G.P., Wilson J.M., Batshaw M.L. (2003). Fatal systemic inflammatory response syndrome in a ornithine transcarbamylase deficient patient following adenoviral gene transfer. Mol. Genet. Metab..

[237] Marshall E. (1999). Gene therapy death prompts review of adenovirus vector. Science.

[238] Hirai H., Satoh E., Osawa M., Inaba T., Shimazaki C., Kinoshita S., Nakagawa M., Mazda O., Imanishi J. (1997). Use of EBV-based Vector/HVJ-liposome complex vector for targeted gene therapy of EBV-associated neoplasms. Biochem. Biophys. Res. Commun..

[239] Caffery B., Lee J.S., Alexander-Bryant A.A. (2019). Vectors for Glioblastoma Gene Therapy: Viral & Non-Viral Delivery Strategies. Nanomaterials.

[240] Han Z., Conley S.M., Makkia R., Guo J., Cooper M.J., Naash M.I. (2012). Comparative Analysis of DNA Nanoparticles and AAVs for Ocular Gene Delivery. PLoS ONE.

[241] Nayerossadat N., Maedeh T., Ali P.A. (2012). Viral and nonviral delivery systems for gene delivery. Adv. Biomed. Res..

[242] Al-Halifa S., Gauthier L., Arpin D., Bourgault S., Archambault D. (2019). Nanoparticle-Based Vaccines Against Respiratory Viruses. Front. Immunol..

[243] Miao X., Leng X., Zhang Q. (2017). The Current State of Nanoparticle-Induced Macrophage Polarization and Reprogramming Research. Int. J. Mol. Sci..

[244] Li J., Liu Y., Xu H., Fu Q. (2016). Nanoparticle-Delivered IRF5 siRNA Facilitates M1 to M2 Transition, Reduces Demyelination and Neurofilament Loss, and Promotes Functional Recovery After Spinal Cord Injury in Mice. Inflammation.

[245] Shobaki N., Sato Y., Suzuki Y., Okabe N., Harashima H. (2020). Manipulating the function of tumor-associated macrophages by siRNA-loaded lipid nanoparticles for cancer immunotherapy. J. Control. Release.

[246] Cecchin R., Troyer Z., Witwer K., Morris K.V. (2023). Extracellular vesicles: The next generation in gene therapy delivery. Mol. Ther..

[247] Liu H., Huang L., Mao M., Ding J., Wu G., Fan W., Yang T., Zhang M., Huang Y., Xie H.-Y. (2020). Viral Protein-Pseudotyped and siRNA-Electroporated Extracellular Vesicles for Cancer Immunotherapy. Adv. Funct. Mater..

[248] Getting S.J. (2002). Melanocortin peptides and their receptors: New targets for anti-inflammatory therapy. Trends Pharmacol. Sci..

[249] Taylor A.W. (2005). The immunomodulating neuropeptide alpha-melanocyte-stimulating hormone (alpha-MSH) suppresses LPS-stimulated TLR4 with IRAK-M in macrophages. J. Neuroimmunol..

[250] Ju N., Hayashi H., Shimamura M., Baba S., Yoshida S., Morishita R., Rakugi H., Nakagami H. (2022). Prevention of bleomycin-induced pulmonary fibrosis by a RANKL peptide in mice. Sci. Rep..

[251] Jha A., Larkin J., Moore E. (2023). SOCS1-KIR Peptide in PEGDA Hydrogels Reduces Pro-Inflammatory Macrophage Activation. Macromol. Biosci..

[252] Zhou W., Kang S., Wang F., Qin Y., Liu J., Xiao X., Chen X., Zhang D. (2023). Chromofungin, a chromogranin A-derived peptide, protects against sepsis-induced acute lung injury by inhibiting LBP/TLR4-dependent inflammatory signaling. Eur. J. Pharmacol..

[253] Gunassekaran G.R., Poongkavithai Vadevoo S.M., Baek M.C., Lee B. (2021). M1 macrophage exosomes engineered to foster M1 polarization and target the IL-4 receptor inhibit tumor growth by reprogramming tumor-associated macrophages into M1-like macrophages. Biomaterials.

[254] Cha B.H., Shin S.R., Leijten J., Li Y.C., Singh S., Liu J.C., Annabi N., Abdi R., Dokmeci M.R., Vrana N.E. (2017). Integrin-Mediated Interactions Control Macrophage Polarization in 3D Hydrogels. Adv. Healthc. Mater..

[255] Lee D., Nah H., Ko W.-K., Jun Kim S., Han G.H., Jeong D., Lee D., Han I., Sheen S.H., Heo D.N. (2022). Thiolate poly(lactic-co-glycolic acid) nanofibers loaded with dexamethasone and ropivacaine show enhanced sustained release in the treatment of neuropathic pain through a local therapy technique. Chem. Eng. J..

[256] Beeraka N.M., Doreswamy S.H., Sadhu S.P., Srinivasan A., Pragada R.R., Madhunapantula S.V., Aliev G. (2020). The Role of Exosomes in Stemness and Neurodegenerative Diseases-Chemoresistant-Cancer Therapeutics and Phytochemicals. Int. J. Mol. Sci..

[257] Sousa A.B., Águas A.P., Barbosa M.A., Barbosa J.N. (2022). Immunomodulatory biomaterial-based wound dressings advance the healing of chronic wounds via regulating macrophage behavior. Regen. Biomater..

[258] Dukhinova M.S., Prilepskii A.Y., Shtil A.A., Vinogradov V.V. (2019). Metal Oxide Nanoparticles in Therapeutic Regulation of Macrophage Functions. Nanomaterials.

[259] Dervan A., Franchi A., Almeida-Gonzalez F.R., Dowling J.K., Kwakyi O.B., McCoy C.E., O’Brien F.J., Hibbitts A. (2021). Biomaterial and Therapeutic Approaches for the Manipulation of Macrophage Phenotype in Peripheral and Central Nerve Repair. Pharmaceutics.

[260] Lohmann N., Schirmer L., Atallah P., Wandel E., Ferrer R.A., Werner C., Simon J.C., Franz S., Freudenberg U. (2017). Glycosaminoglycan-based hydrogels capture inflammatory chemokines and rescue defective wound healing in mice. Sci. Transl. Med..

[261] Rezvani Ghomi E., Nourbakhsh N., Akbari Kenari M., Zare M., Ramakrishna S. (2021). Collagen-based biomaterials for biomedical applications. J. Biomed. Mater. Res. Part B Appl. Biomater..

[262] Fakhri E., Eslami H., Maroufi P., Pakdel F., Taghizadeh S., Ganbarov K., Yousefi M., Tanomand A., Yousefi B., Mahmoudi S. (2020). Chitosan biomaterials application in dentistry. Int. J. Biol. Macromol..

[263] Yang K., Zhou C., Fan H., Fan Y., Jiang Q., Song P., Fan H., Chen Y., Zhang X. (2018). Bio-Functional Design, Application and Trends in Metallic Biomaterials. Int. J. Mol. Sci..

[264] Eliaz N. (2019). Corrosion of Metallic Biomaterials: A Review. Materials.

[265] Choi S.-r., Kwon J.-w., Suk K.-s., Kim H.-s., Moon S.-h., Park S.-y., Lee B.H. (2023). The Clinical Use of Osteobiologic and Metallic Biomaterials in Orthopedic Surgery: The Present and the Future. Materials.

[266] Lima F.d.S., Fock R.A. (2020). A Review of the Action of Magnesium on Several Processes Involved in the Modulation of Hematopoiesis. Int. J. Mol. Sci..

[267] Kazakova G., Safronova T., Golubchikov D., Shevtsova O., Rau J.V. (2021). Resorbable Mg2+-containing phosphates for bone tissue repair. Materials.

[268] Figueiredo Borgognoni C., Kim J.H., Zucolotto V., Fuchs H., Riehemann K. (2018). Human macrophage responses to metal-oxide nanoparticles: A review. Artif. Cells Nanomed. Biotechnol..

[269] Nagajyothi P.C., Cha S.J., Yang I.J., Sreekanth T.V., Kim K.J., Shin H.M. (2015). Antioxidant and anti-inflammatory activities of zinc oxide nanoparticles synthesized using Polygala tenuifolia root extract. J. Photochem. Photobiol. B.

[270] Antonoglou O., Lafazanis K., Mourdikoudis S., Vourlias G., Lialiaris T., Pantazaki A., Dendrinou-Samara C. (2019). Biological relevance of CuFeO(2) nanoparticles: Antibacterial and anti-inflammatory activity, genotoxicity, DNA and protein interactions. Mater. Sci. Eng. C Mater. Biol. Appl..

[271] Ali S.S., Morsy R., El-Zawawy N.A., Fareed M.F., Bedaiwy M.Y. (2017). Synthesized zinc peroxide nanoparticles (ZnO(2)-NPs): A novel antimicrobial, anti-elastase, anti-keratinase, and anti-inflammatory approach toward polymicrobial burn wounds. Int. J. Nanomed..

[272] Seisenbaeva G.A., Fromell K., Vinogradov V.V., Terekhov A.N., Pakhomov A.V., Nilsson B., Ekdahl K.N., Vinogradov V.V., Kessler V.G. (2017). Dispersion of TiO(2) nanoparticles improves burn wound healing and tissue regeneration through specific interaction with blood serum proteins. Sci. Rep..

[273] Serebrovska Z., Swanson R.J., Portnichenko V., Shysh A., Pavlovich S., Tumanovska L., Dorovskych A., Lysenko V., Tertykh V., Bolbukh Y. (2017). Anti-inflammatory and antioxidant effect of cerium dioxide nanoparticles immobilized on the surface of silica nanoparticles in rat experimental pneumonia. Biomed. Pharmacother..

[274] Heckman K.L., DeCoteau W., Estevez A., Reed K.J., Costanzo W., Sanford D., Leiter J.C., Clauss J., Knapp K., Gomez C. (2013). Custom cerium oxide nanoparticles protect against a free radical mediated autoimmune degenerative disease in the brain. ACS Nano.

[275] Song Y., You Q., Chen X. (2023). Transition Metal-Based Therapies for Inflammatory Diseases. Adv. Mater..

[276] Wang Y., Fan Y., Liu H. (2021). Macrophage Polarization in Response to Biomaterials for Vascularization. Ann. Biomed. Eng..

[277] Torregrossa M., Kakpenova A., Simon J.C., Franz S. (2021). Modulation of macrophage functions by ECM-inspired wound dressings—A promising therapeutic approach for chronic wounds. Biol. Chem..

[278] Huleihel L., Dziki J.L., Bartolacci J.G., Rausch T., Scarritt M.E., Cramer M.C., Vorobyov T., LoPresti S.T., Swineheart I.T., White L.J. (2017). Macrophage phenotype in response to ECM bioscaffolds. Semin. Immunol..

[279] Elhadad A.A., Alcudia A., Begines B., Pérez-Soriano E.M., Torres Y. (2022). A multidisciplinary perspective on the latest trends in artificial cartilage fabrication to mimic real tissue. Appl. Mater. Today.

[280] Shoulders M.D., Raines R.T. (2009). Collagen Structure and Stability. Annu. Rev. Biochem..

[281] Belda Marín C., Fitzpatrick V., Kaplan D.L., Landoulsi J., Guénin E., Egles C. (2020). Silk Polymers and Nanoparticles: A Powerful Combination for the Design of Versatile Biomaterials. Front. Chem..

[282] Ghatak S., Maytin E.V., Mack J.A., Hascall V.C., Atanelishvili I., Moreno Rodriguez R., Markwald R.R., Misra S. (2015). Roles of Proteoglycans and Glycosaminoglycans in Wound Healing and Fibrosis. Int. J. Cell Biol..

[283] Berdiaki A., Neagu M., Giatagana E.-M., Kuskov A., Tsatsakis A.M., Tzanakakis G.N., Nikitovic D. (2021). Glycosaminoglycans: Carriers and Targets for Tailored Anti-Cancer Therapy. Biomolecules.

[284] Litwiniuk M., Krejner A., Speyrer M.S., Gauto A.R., Grzela T. (2016). Hyaluronic acid in inflammation and tissue regeneration. Wounds.

[285] Snetkov P., Zakharova K., Morozkina S., Olekhnovich R., Uspenskaya M. (2020). Hyaluronic Acid: The Influence of Molecular Weight on Structural, Physical, Physico-Chemical, and Degradable Properties of Biopolymer. Polymers.

[286] Stern R., Asari A.A., Sugahara K.N. (2006). Hyaluronan fragments: An information-rich system. Eur. J. Cell Biol..

[287] Hintze V., Schnabelrauch M., Rother S. (2022). Chemical modification of hyaluronan and their biomedical applications. Front. Chem..

[288] Zhang M., James S. (2005). Synthesis and properties of melt-processable hyaluronan esters. J. Mater. Sci..

[289] Kawaguchi Y., Matsukawa K., Ishigami Y. (1995). The relation between the adsorption behavior at the interface and the conformational changes in hyaluronates partially modified with various acyl chains. Carbohydr. Polym..

[290] Zhang R., Huang Z., Xue M., Yang J., Tan T. (2011). Detailed characterization of an injectable hyaluronic acid-polyaspartylhydrazide hydrogel for protein delivery. Carbohydr. Polym..

[291] Dahl L.B., Laurent T.C., Smedsrød B. (1988). Preparation of biologically intact radioiodinated hyaluronan of high specific radioactivity: Coupling of 125I-tyramine-cellobiose to amino groups after partial N-deacetylation. Anal. Biochem..

[292] Balazs E., Högberg B., Laurent T. (1951). The biological activity of hyaluron sulfuric acid. Acta Physiol. Scand..

[293] Magnani A., Albanese A., Lamponi S., Barbucci R. (1996). Blood-interaction performance of differently sulphated hyaluronic acids. Thromb. Res..

[294] Christgen S., Place D.E., Kanneganti T.D. (2020). Toward targeting inflammasomes: Insights into their regulation and activation. Cell Res..

[295] Jouy F., Lohmann N., Wandel E., Ruiz-Gómez G., Pisabarro M.T., Beck-Sickinger A.G., Schnabelrauch M., Möller S., Simon J.C., Kalkhof S. (2017). Sulfated hyaluronan attenuates inflammatory signaling pathways in macrophages involving induction of antioxidants. Proteomics.

[296] Hauck S., Zager P., Halfter N., Wandel E., Torregrossa M., Kakpenova A., Rother S., Ordieres M., Räthel S., Berg A. (2021). Collagen/hyaluronan based hydrogels releasing sulfated hyaluronan improve dermal wound healing in diabetic mice via reducing inflammatory macrophage activity. Bioact. Mater..

[297] Hatano S., Watanabe H. (2020). Regulation of Macrophage and Dendritic Cell Function by Chondroitin Sulfate in Innate to Antigen-Specific Adaptive Immunity. Front. Immunol..

[298] Couchman J.R., Pataki C.A. (2012). An introduction to proteoglycans and their localization. J. Histochem. Cytochem..

[299] Taylor K.R., Gallo R.L. (2006). Glycosaminoglycans and their proteoglycans: Host-associated molecular patterns for initiation and modulation of inflammation. FASEB J.

[300] Hascall V.C., Majors A.K., De La Motte C.A., Evanko S.P., Wang A., Drazba J.A., Strong S.A., Wight T.N. (2004). Intracellular hyaluronan: A new frontier for inflammation?. Biochim. Biophys. Acta.

[301] Tremmel M., Matzke A., Albrecht I., Laib A.M., Olaku V., Ballmer-Hofer K., Christofori G., Héroult M., Augustin H.G., Ponta H. (2009). A CD44v6 peptide reveals a role of CD44 in VEGFR-2 signaling and angiogenesis. Blood.

[302] Rayahin J.E., Buhrman J.S., Zhang Y., Koh T.J., Gemeinhart R.A. (2015). High and low molecular weight hyaluronic acid differentially influence macrophage activation. ACS Biomater. Sci. Eng..

[303] Zhao J., Feng Y., Liu X., Li H., Guo H., Ke J., Long X. (2024). The relationship of ALPK1, hyaluronic acid and M1 macrophage polarization in the temporomandibular joint synovitis. J. Cell. Mol. Med..

[304] Gao Y., Sun Y., Yang H., Qiu P., Cong Z., Zou Y., Song L., Guo J., Anastassiades T.P. (2019). A Low Molecular Weight Hyaluronic Acid Derivative Accelerates Excisional Wound Healing by Modulating Pro-Inflammation, Promoting Epithelialization and Neovascularization, and Remodeling Collagen. Int. J. Mol. Sci..

[305] Jiang D., Liang J., Fan J., Yu S., Chen S., Luo Y., Prestwich G.D., Mascarenhas M.M., Garg H.G., Quinn D.A. (2005). Regulation of lung injury and repair by Toll-like receptors and hyaluronan. Nat. Med..

[306] Petrey A.C., de la Motte C.A. (2014). Hyaluronan, a crucial regulator of inflammation. Front. Immunol..

[307] Kobayashi T., Chanmee T., Itano N. (2020). Hyaluronan: Metabolism and Function. Biomolecules.

[308] Shi Q., Zhao L., Xu C., Zhang L., Zhao H. (2019). High Molecular Weight Hyaluronan Suppresses Macrophage M1 Polarization and Enhances IL-10 Production in PM2.5-Induced Lung Inflammation. Molecules.

[309] Lee B.M., Park S.J., Noh I., Kim C.-H. (2021). The effects of the molecular weights of hyaluronic acid on the immune responses. Biomater. Res..

[310] Lee C.-H., Chiang C.-F., Kuo F.-C., Su S.-C., Huang C.-L., Liu J.-S., Lu C.-H., Hsieh C.-H., Wang C.-C., Lee C.-H. (2021). High-Molecular-Weight Hyaluronic Acid Inhibits IL-1β-Induced Synovial Inflammation and Macrophage Polarization through the GRP78-NF-κB Signaling Pathway. Int. J. Mol. Sci..

[311] Xu C.X., Jin H., Chung Y.S., Shin J.Y., Woo M.A., Lee K.H., Palmos G.N., Choi B.D., Cho M.H. (2008). Chondroitin sulfate extracted from the Styela clava tunic suppresses TNF-alpha-induced expression of inflammatory factors, VCAM-1 and iNOS by blocking Akt/NF-kappaB signal in JB6 cells. Cancer Lett..

[312] Jomphe C., Gabriac M., Hale T.M., Héroux L., Trudeau L.E., Deblois D., Montell E., Vergés J., du Souich P. (2008). Chondroitin sulfate inhibits the nuclear translocation of nuclear factor-kappaB in interleukin-1beta-stimulated chondrocytes. Basic. Clin. Pharmacol. Toxicol..

[313] Zhang W., Sun F., Niu H., Wang Q., Duan J. (2015). Mechanistic insights into cellular immunity of chondroitin sulfate A and its zwitterionic N-deacetylated derivatives. Carbohydr. Polym..

[314] Campo G.M., Avenoso A., Campo S., Traina P., D’Ascola A., Calatroni A. (2009). Glycosaminoglycans reduced inflammatory response by modulating toll-like receptor-4 in LPS-stimulated chondrocytes. Arch. Biochem. Biophys..

[315] Kastana P., Choleva E., Poimenidi E., Karamanos N., Sugahara K., Papadimitriou E. (2019). Insight into the role of chondroitin sulfate E in angiogenesis. FEBS J..

[316] Taraballi F., Corradetti B., Minardi S., Powel S., Cabrera F., Van Eps J.L., Weiner B.K., Tasciotti E. (2016). Biomimetic collagenous scaffold to tune inflammation by targeting macrophages. J. Tissue Eng..

[317] Tan G.K., Tabata Y. (2014). Chondroitin-6-sulfate attenuates inflammatory responses in murine macrophages via suppression of NF-κB nuclear translocation. Acta Biomater..

[318] Pudełko A., Wisowski G., Olczyk K., Koźma E.M. (2019). The dual role of the glycosaminoglycan chondroitin-6-sulfate in the development, progression and metastasis of cancer. FEBS J.

[319] Coombe D.R. (2008). Biological implications of glycosaminoglycan interactions with haemopoietic cytokines. Immunol. Cell Biol..

[320] Hasan M., Najjam S., Gordon M.Y., Gibbs R.V., Rider C.C. (1999). IL-12 is a heparin-binding cytokine. J. Immunol..

[321] Esko J.D., Lindahl U. (2001). Molecular diversity of heparan sulfate. J. Clin. Investig..

[322] Nelson S.M., Greer I.A. (2008). The potential role of heparin in assisted conception. Hum. Reprod. Update.

[323] Page C. (2013). Heparin and related drugs: Beyond anticoagulant activity. ISRN Pharmacol..

[324] Vivès R.R., Sadir R., Imberty A., Rencurosi A., Lortat-Jacob H. (2002). A kinetics and modeling study of RANTES(9-68) binding to heparin reveals a mechanism of cooperative oligomerization. Biochemistry.

[325] Li J.P., Vlodavsky I. (2009). Heparin, heparan sulfate and heparanase in inflammatory reactions. Thromb. Haemost..

[326] Stringer S.E., Nelson M.S., Gupta P. (2003). Identification of an MIP-1alpha -binding heparan sulfate oligosaccharide that supports long-term in vitro maintenance of human LTC-ICs. Blood.

[327] Zhu M., Wu X., Sun J., Zhou Z., Kang M., Hu Y., Teng L. (2023). N-desulfated and reacetylated modification of heparin modulates macrophage polarization. Int. J. Biol. Macromol..

[328] Gordts P., Foley E.M., Lawrence R., Sinha R., Lameda-Diaz C., Deng L., Nock R., Glass C.K., Erbilgin A., Lusis A.J. (2014). Reducing macrophage proteoglycan sulfation increases atherosclerosis and obesity through enhanced type I interferon signaling. Cell Metab..

[329] Funderburgh J.L. (2000). MINI REVIEW Keratan sulfate: Structure, biosynthesis, and function. Glycobiology.

[330] Funderburgh J.L. (2002). Keratan sulfate biosynthesis. IUBMB Life.

[331] Caterson B., Melrose J. (2018). Keratan sulfate, a complex glycosaminoglycan with unique functional capability. Glycobiology.

[332] Hayashi M., Kadomatsu K., Kojima T., Ishiguro N. (2011). Keratan sulfate and related murine glycosylation can suppress murine cartilage damage in vitro and in vivo. Biochem. Biophys. Res. Commun..

[333] Xu H., Kurihara H., Ito T., Kikuchi H., Yoshida K., Yamanokuchi H., Asari A. (2005). The keratan sulfate disaccharide Gal(6S03) beta1,4-GlcNAc(6S03) modulates interleukin 12 production by macrophages in murine Thy-1 type autoimmune disease. J. Biol. Chem..

[334] Sharma R., Kuche K., Thakor P., Bhavana V., Srivastava S., Mehra N.K., Jain S. (2022). Chondroitin Sulfate: Emerging biomaterial for biopharmaceutical purpose and tissue engineering. Carbohydr. Polym..

[335] Kwon H., Han Y. (2016). Chondroitin sulfate-based biomaterials for tissue engineering. Turk. J. Biol..

[336] Li C., Wang K., Zhou X., Li T., Xu Y., Qiang L., Peng M., Xu Y., Xie L., He C. (2019). Controllable fabrication of hydroxybutyl chitosan/oxidized chondroitin sulfate hydrogels by 3D bioprinting technique for cartilage tissue engineering. Biomed. Mater..

[337] Ashikari-Hada S., Habuchi H., Kariya Y., Kimata K. (2005). Heparin Regulates Vascular Endothelial Growth Factor165-dependent Mitogenic Activity, Tube Formation, and Its Receptor Phosphorylation of Human Endothelial Cells: Comparison of the Effects of Heparin and Modified Heparins*. J. Biol. Chem..

[338] Rabenstein D.L. (2002). Heparin and heparan sulfate: Structure and function. Nat. Prod. Rep..

[339] Liang Y., Kiick K.L. (2014). Heparin-functionalized polymeric biomaterials in tissue engineering and drug delivery applications. Acta Biomater..

[340] Feng Y., Li Q., Wu D., Niu Y., Yang C., Dong L., Wang C. (2017). A macrophage-activating, injectable hydrogel to sequester endogenous growth factors for in situ angiogenesis. Biomaterials.

[341] Belvedere R., Novizio N., Palazzo M., Pessolano E., Petrella A. (2023). The pro-healing effects of heparan sulfate and growth factors are enhanced by the heparinase enzyme: New association for skin wound healing treatment. Eur. J. Pharmacol..

[342] Devernois E., Coradin T. (2023). Synthesis, Characterization and Biological Properties of Type I Collagen–Chitosan Mixed Hydrogels: A Review. Gels.

[343] Negm N.A., Hefni H.H.H., Abd-Elaal A.A.A., Badr E.A., Abou Kana M.T.H. (2020). Advancement on modification of chitosan biopolymer and its potential applications. Int. J. Biol. Macromol..

[344] von Boxberg Y., Soares S., Giraudon C., David L., Viallon M., Montembault A., Nothias F. (2022). Macrophage polarization in vitro and in vivo modified by contact with fragmented chitosan hydrogel. J. Biomed. Mater. Res. Part A.

[345] Oliveira M.I., Santos S.G., Oliveira M.J., Torres A.L., Barbosa M.A. (2012). Chitosan drives anti-inflammatory macrophage polarisation and pro-inflammatory dendritic cell stimulation. Eur. Cell Mater..

[346] Shen T., Dai K., Yu Y., Wang J., Liu C. (2020). Sulfated chitosan rescues dysfunctional macrophages and accelerates wound healing in diabetic mice. Acta Biomater..

[347] You C., Zhu Z., Wang S., Wang X., Han C., Shao H. (2023). Nanosilver alleviates foreign body reaction and facilitates wound repair by regulating macrophage polarization. J. Zhejiang Univ. Sci. B.

[348] You C., Li Q., Wang X., Wu P., Ho J.K., Jin R., Zhang L., Shao H., Han C. (2017). Silver nanoparticle loaded collagen/chitosan scaffolds promote wound healing via regulating fibroblast migration and macrophage activation. Sci. Rep..

[349] You C., Han C., Wang X., Zheng Y., Li Q., Hu X., Sun H. (2012). The progress of silver nanoparticles in the antibacterial mechanism, clinical application and cytotoxicity. Mol. Biol. Rep..

[350] Bhol K., Schechter P. (2005). Topical nanocrystalline silver cream suppresses inflammatory cytokines and induces apoptosis of inflammatory cells in a murine model of allergic contact dermatitis. Br. J. Dermatol..

[351] Gil E.S., Panilaitis B., Bellas E., Kaplan D.L. (2013). Functionalized silk biomaterials for wound healing. Adv. Healthc. Mater..

[352] Li X., Liu Y., Zhang J., You R., Qu J., Li M. (2017). Functionalized silk fibroin dressing with topical bioactive insulin release for accelerated chronic wound healing. Mater. Sci. Eng. C.

[353] Wang Y., Yao D., Li L., Qian Z., He W., Ding R., Liu H., Fan Y. (2020). Effect of electrospun silk fibroin–silk sericin films on macrophage polarization and vascularization. ACS Biomater. Sci. Eng..

[354] Roy S., Sharma A., Ghosh S. (2022). Macrophage polarization profiling on native and regenerated silk biomaterials. ACS Biomater. Sci. Eng..

[355] Reeves A.R., Spiller K.L., Freytes D.O., Vunjak-Novakovic G., Kaplan D.L. (2015). Controlled release of cytokines using silk-biomaterials for macrophage polarization. Biomaterials.

[356] Reddy M.S.B., Ponnamma D., Choudhary R., Sadasivuni K.K. (2021). A Comparative Review of Natural and Synthetic Biopolymer Composite Scaffolds. Polymers.

[357] Abbasian M., Massoumi B., Mohammad-Rezaei R., Samadian H., Jaymand M. (2019). Scaffolding polymeric biomaterials: Are naturally occurring biological macromolecules more appropriate for tissue engineering?. Int. J. Biol. Macromol..

[358] Kyriakides T.R., Kim H.J., Zheng C., Harkins L., Tao W., Deschenes E. (2022). Foreign body response to synthetic polymer biomaterials and the role of adaptive immunity. Biomed. Mater..

[359] Mir M., Ali M.N., Barakullah A., Gulzar A., Arshad M., Fatima S., Asad M. (2018). Synthetic polymeric biomaterials for wound healing: A review. Progress. Biomater..

[360] Liu X., Chen M., Luo J., Zhao H., Zhou X., Gu Q., Yang H., Zhu X., Cui W., Shi Q. (2021). Immunopolarization-regulated 3D printed-electrospun fibrous scaffolds for bone regeneration. Biomaterials.

[361] Nakkala J.R., Duan Y., Ding J., Muhammad W., Zhang D., Mao Z., Ouyang H., Gao C. (2022). Macrophage membrane-functionalized nanofibrous mats and their immunomodulatory effects on macrophage polarization. Acta Biomater..

[362] Zhang Q., Chen J., Lin J., Liang R., He M., Wang Y., Tan H. (2023). Porous Three-Dimensional Polyurethane Scaffolds Promote Scar-Free Endogenous Regeneration After Acute Brain Hemorrhage. Transl. Stroke Res..

[363] Wolf M.T., Dearth C.L., Ranallo C.A., LoPresti S.T., Carey L.E., Daly K.A., Brown B.N., Badylak S.F. (2014). Macrophage polarization in response to ECM coated polypropylene mesh. Biomaterials.

[364] Li Y.-M., Wu J.-Y., Jiang J., Dong S.-K., Chen Y.-S., He H.-Y., Liu C.-S., Zhao J.-Z. (2019). Chondroitin sulfate-polydopamine modified polyethylene terephthalate with extracellular matrix-mimetic immunoregulatory functions for osseointegration. J. Mater. Chem. B.

[365] Hook A.L., Anderson D.G., Langer R., Williams P., Davies M.C., Alexander M.R. (2010). High throughput methods applied in biomaterial development and discovery. Biomaterials.

[366] Crawford L.A., Cuzzucoli Crucitti V., Stimpson A., Morgan C., Blake J., Wildman R.D., Hook A.L., Alexander M.R., Irvine D.J., Avery S.V. (2023). A potential alternative to fungicides using actives-free (meth)acrylate polymers for protection of wheat crops from fungal attachment and infection. Green. Chem..

[367] Wong S.Y., Hook A.L., Gardner W., Chang C.-Y., Mei Y., Davies M.C., Williams P., Alexander M.R., Ballabio D., Muir B.W. (2023). Exploring the Relationship between Polymer Surface Chemistry and Bacterial Attachment Using ToF-SIMS and Self-Organizing maps. Adv. Mater. Interfaces.

[368] Vallieres C., Hook A.L., He Y., Crucitti V.C., Figueredo G., Davies C.R., Burroughs L., Winkler D.A., Wildman R.D., Irvine D.J. (2020). Discovery of (meth)acrylate polymers that resist colonization by fungi associated with pathogenesis and biodeterioration. Sci. Adv..

[369] Rostam H.M., Fisher L.E., Hook A.L., Burroughs L., Luckett J.C., Figueredo G.P., Mbadugha C., Teo A.C., Latif A., Kämmerling L. (2020). Immune-instructive polymers control macrophage phenotype and modulate the foreign body response in vivo. Matter.

[370] Latif A., Fisher L.E., Dundas A.A., Cuzzucoli Crucitti V., Imir Z., Lawler K., Pappalardo F., Muir B.W., Wildman R., Irvine D.J. (2022). Microparticles Decorated with Cell-Instructive Surface Chemistries Actively Promote Wound Healing. Adv. Mater..

[371] Luu T.U., Gott S.C., Woo B.W., Rao M.P., Liu W.F. (2015). Micro-and nanopatterned topographical cues for regulating macrophage cell shape and phenotype. ACS Appl. Mater. Interfaces.

[372] Vassey M.J., Figueredo G.P., Scurr D.J., Vasilevich A.S., Vermeulen S., Carlier A., Luckett J., Beijer N.R., Williams P., Winkler D.A. (2020). Immune modulation by design: Using topography to control human monocyte attachment and macrophage differentiation. Adv. Sci..

[373] Monteiro N., Casanova M., Quinteira R., Fangueiro J., Reis R., Neves N. (2022). Biomimetic surface topography as a potential modulator of macrophages inflammatory response to biomaterials. Biomater. Adv..

[374] Singh S., Awuah D., Rostam H.M., Emes R.D., Kandola N.K., Onion D., Htwe S.S., Rajchagool B., Cha B.-H., Kim D. (2017). Unbiased analysis of the impact of micropatterned biomaterials on macrophage behavior provides insights beyond predefined polarization states. ACS Biomater. Sci. Eng..

[375] Jia Y., Yang W., Zhang K., Qiu S., Xu J., Wang C., Chai Y. (2019). Nanofiber arrangement regulates peripheral nerve regeneration through differential modulation of macrophage phenotypes. Acta Biomater..

[376] Han D., Gouma P.-I. (2006). Electrospun bioscaffolds that mimic the topology of extracellular matrix. Nanomed. Nanotechnol. Biol. Med..

[377] Hotchkiss K.M., Reddy G.B., Hyzy S.L., Schwartz Z., Boyan B.D., Olivares-Navarrete R. (2016). Titanium surface characteristics, including topography and wettability, alter macrophage activation. Acta Biomater..

[378] Duan Y., Zheng H., Li Z., Yao Y., Ding J., Wang X., Nakkala J.R., Zhang D., Wang Z., Zuo X. (2020). Unsaturated polyurethane films grafted with enantiomeric polylysine promotes macrophage polarization to a M2 phenotype through PI3K/Akt1/mTOR axis. Biomaterials.

[379] Huang Y.-J., Hung K.-C., Hung H.-S., Hsu S.-h. (2018). Modulation of macrophage phenotype by biodegradable polyurethane nanoparticles: Possible relation between macrophage polarization and immune response of nanoparticles. ACS Appl. Mater. Interfaces.

[380] Bartneck M., Keul H.A., Singh S., Czaja K., Bornemann J., Bockstaller M., Moeller M., Zwadlo-Klarwasser G., Groll J. (2010). Rapid uptake of gold nanorods by primary human blood phagocytes and immunomodulatory effects of surface chemistry. ACS Nano.

[381] Jones J.A., Chang D.T., Meyerson H., Colton E., Kwon I.K., Matsuda T., Anderson J.M. (2007). Proteomic analysis and quantification of cytokines and chemokines from biomaterial surface-adherent macrophages and foreign body giant cells. J. Biomed. Mater. Res. Part A.

[382] Scott R.A., Kiick K.L., Akins R.E. (2021). Substrate stiffness directs the phenotype and polarization state of cord blood derived macrophages. Acta Biomater..

[383] Chen M., Zhang Y., Zhou P., Liu X., Zhao H., Zhou X., Gu Q., Li B., Zhu X., Shi Q. (2020). Substrate stiffness modulates bone marrow-derived macrophage polarization through NF-κB signaling pathway. Bioact. Mater..

[384] Camarero-Espinosa S., Carlos-Oliveira M., Liu H., Mano J.F., Bouvy N., Moroni L. (2022). 3D printed dual-porosity scaffolds: The combined effect of stiffness and porosity in the modulation of macrophage polarization. Adv. Healthc. Mater..

[385] Roach P., Eglin D., Rohde K., Perry C.C. (2007). Modern biomaterials: A review—Bulk properties and implications of surface modifications. J. Mater. Sci. Mater. Med..

